# Scalable microfabrication of monolithic integrated microbatteries with ultra-high voltage output and excellent customizability

**DOI:** 10.1093/nsr/nwaf302

**Published:** 2025-07-28

**Authors:** Yuan Ma, Sen Wang, Zhuobin Guo, Xiao Wang, Yuxin Ma, Yinghua Fu, Hanqing Liu, Shengwei Li, Yao Lu, Zhizhang Yuan, Zhong-Shuai Wu

**Affiliations:** State Key Laboratory of Catalysis, Dalian Institute of Chemical Physics, Chinese Academy of Sciences, Dalian 116023, China; University of Chinese Academy of Sciences, Beijing 100049, China; State Key Laboratory of Catalysis, Dalian Institute of Chemical Physics, Chinese Academy of Sciences, Dalian 116023, China; School of Transportation Engineering, Dalian Jiaotong University, Dalian 116028, China; State Key Laboratory of Catalysis, Dalian Institute of Chemical Physics, Chinese Academy of Sciences, Dalian 116023, China; University of Chinese Academy of Sciences, Beijing 100049, China; State Key Laboratory of Catalysis, Dalian Institute of Chemical Physics, Chinese Academy of Sciences, Dalian 116023, China; State Key Laboratory of Catalysis, Dalian Institute of Chemical Physics, Chinese Academy of Sciences, Dalian 116023, China; University of Chinese Academy of Sciences, Beijing 100049, China; State Key Laboratory of Catalysis, Dalian Institute of Chemical Physics, Chinese Academy of Sciences, Dalian 116023, China; University of Chinese Academy of Sciences, Beijing 100049, China; State Key Laboratory of Catalysis, Dalian Institute of Chemical Physics, Chinese Academy of Sciences, Dalian 116023, China; University of Chinese Academy of Sciences, Beijing 100049, China; State Key Laboratory of Catalysis, Dalian Institute of Chemical Physics, Chinese Academy of Sciences, Dalian 116023, China; State Key Laboratory of Phytochemistry and Natural Medicines, Dalian Institute of Chemical Physics, Chinese Academy of Sciences, Dalian 116023, China; Division of Energy Storage, Dalian National Laboratory for Clean Energy, Dalian Institute of Chemical Physics, Chinese Academy of Sciences, Dalian 116023, China; State Key Laboratory of Catalysis, Dalian Institute of Chemical Physics, Chinese Academy of Sciences, Dalian 116023, China

**Keywords:** microbatteries, photolithography, monolithic integration, ultra-high voltage output

## Abstract

The burgeoning Internet of Things demands highly customizable microbatteries (MBs) to power miniaturized electronics, yet challenges exist in fabricating ultra-small MBs and integrating customizable modules within confined areas. Herein, we report a novel photolithographic microfabrication strategy enabling the large-scale production of monolithic integrated ultra-small MBs. The approach utilizes photoresist grooves as micropattern templates and employs a non-destructive mechanical peeling process to fabricate precise MBs with a compact area of 2.2275 mm^2^ by using Li_3_V_2_(PO_4_)_3_ as both the cathode and the anode. These MBs demonstrate an exceptional areal capacity of 96.4 μAh cm^−2^ and remarkable cycling stability, retaining 88.3% of their initial capacity after 10 000 cycles. Furthermore, the method allows the facile serial integration of numerous MBs in a single step, achieving a record voltage of 182.7 V through 63 series-connected units. This breakthrough provides a scalable solution for mass-producing customizable MBs, advancing the power supply capabilities for miniature electronics with high energy density and long-term reliability.

## Teaser

Scalable microfabrication of integrated microbatteries with excellent stability and ultrahigh voltage output is achieved by non-destructively peeling of photoresist template.

## INTRODUCTION

The rapid development of Internet of Things (IoT) technology has vigorously stimulated the boom in miniaturized electronic devices (MEDs), such as microelectromechanical systems, microsensors, intelligent electronic equipment, microrobots and implantable medical devices [1–7]. Such MEDs are usually millimeters or even microns in size, which highly require micro energy-storage devices (MESDs) that can supply steady power at the nW∼mW level and possess the corresponding size for on-chip seamless integration [8–14]. Micro-supercapacitors (MSCs) and microbatteries (MBs) are the two most commonly used MESDs, in which the MBs have much higher energy density through the bulky Faradaic process mechanism compared with the surface adsorption and desorption of ions or surface pseudocapacitive process in MSCs [1,3,4,15] and hold great promise as advanced MESDs. However, the complexity of the electrochemical system of the MBs causes many difficulties in the fabrication of the MBs regarding materials selection, configuration design and fabrication technology, making the development of MBs lag far behind the requirements of current MEDs [10].

The design of symmetric MBs with identical materials serving dual roles as both cathode and anode offers a promising solution to circumvent the abovementioned issues associated with traditional battery designs. This design demonstrates significant advantages, particularly in planar MBs, in which the cathode and anode electrodes are located on the same substrate and made of the same material, allowing simultaneous construction. It can significantly simplify the complexity of the manufacturing process, shorten the preparation workflow and effectively reduce costs. From a commercial perspective, this innovative design is highly attractive and holds broad prospects for development. In addition, introducing symmetric configuration into MBs can efficiently improve their safety and stability. Most of the active materials involved in symmetric batteries (e.g. Li_3_V_2_(PO_4_)_3_ (LVP) [16], LiVPO_4_F [17], Na_3_V_2_(PO_4_)_3_ [18], Na_3_V_2_(PO_4_)_2_F_3_ [19], Na_2_VTi(PO_4_)_3_ [20]) undergo the highly reversible insertion/deinsertion of ions during charging and discharging processes, and the intricate side reactions or even dendrites brought by the alloy-type or metal anode can be effectively circumvented, which improves the safety and cycling life of MBs [21].

As for the specific microfabrication methods of MBs, photolithography possesses the highest resolution at the micron level, mature development and the ability to customize patterns, making it a promising fabrication technology for MBs to keep pace with the ever-shrinking MEDs. Nevertheless, precisely loading the electrode materials onto micropatterns prepared by photolithography poses a significant challenge due to the tiny electrode area, resulting in the risk of short circuit between the cathode and the anode. Conformal deposition technologies, such as electrodeposition [22–25] and vapor deposition [26–29], are commonly used to circumvent the above difficulties. However, conformal deposition technologies are often inefficient and the prepared electrode films tend to be dense with residual stress, making it very difficult to achieve thick electrodes with high areal capacity. Although 3D current collectors have been introduced to increase the mass loading of the electrode materials [1,3], the intricate fabrication process of the 3D current collector also reduces productivity. In addition, such conformal deposition technologies can hardly achieve the integrated fabrication of multiple MBs through a simple process and are unfavorable for catering to the high voltage requirement that necessitates serial integration. Furthermore, some researchers have directly utilized patterned materials, such as SU-8 photoresist [30] and poly(dimethylsiloxane) (PDMS) [31], as grooves for the microelectrodes, enabling the construction of thick electrodes with high mass loading. However, the grooves used are not conductive to electrolyte ions and are difficult to remove, resulting in poor rate performance of the MBs. Therefore, developing an efficient, universal and scalable strategy for fabricating high-capacity, high-rate and high-precision MBs based on photolithography technology holds significant scientific importance and practical value.

Here, we have developed a non-destructive peeling strategy for the patterned photoresist, enabling efficient manufacturing of ultra-miniaturized microelectrodes and the mass production of monolithic integrated MBs on a large scale. It was achieved by creating a patterned photoresist groove using photolithography technology and the photoresist groove was used as the template to define the patterns of the LVP microelectrodes serving as both cathode and anode. Combined with gentle heating and a straightforward mechanical peeling process, the photoresist could be entirely and non-destructively removed due to the significant difference in the coefficients of thermal expansion between the photoresist and the substrate, producing high-precision microelectrodes and integrated modules. Using the ionic liquid electrolyte, the as-fabricated MBs could deliver a decent areal energy density of 195.5 μWh cm^−2^ and an incredible capacity retention of 88.3% after long-term cycling of 10 000 times. It was revealed that the excellent cycling stability resulted from the highly reversible structural evolution of LVP during charge and discharge, compatible non-destructive microfabrication technique and control over the thickness/quality in the microfabrication process. Furthermore, taking advantage of the customizability of photolithography, our strategy could conveniently realize the serial integration of a large number of MBs and reach a record-high voltage of 182.7 V.

## RESULTS AND DISCUSSION

**Figure 1.**
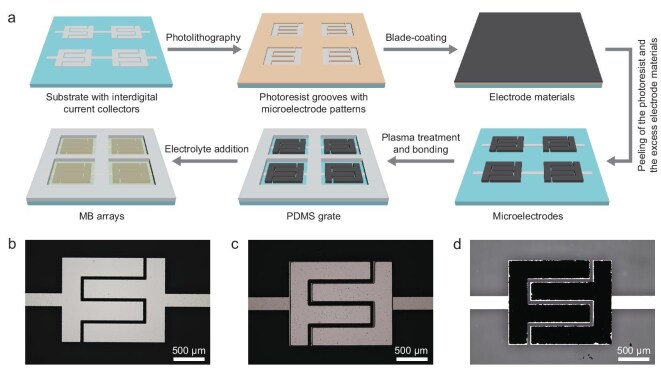
Schematic illustration and optical images depicting the microfabrication steps of monolithic integrated MBs. (a) Schematic illustration of the monolithic integrated MBs fabrication process, including fabricating interdigital current collectors, constructing photoresist grooves, blade-coating of the electrode material, removing the photoresist grooves, bonding the PDMS grate and adding the electrolyte. Optical microscope images of (b) the interdigital current collector, (c) the SU-8 photoresist grooves and (d) the microelectrode on the current collector with two fingers on one side.

The schematic illustration of the monolithic integrated MB microfabrication process is depicted in Fig. 1a (see details in Experimental Section and Fig. S1 in the online Supplementary file). The Ti/Al interdigital current collectors for microelectrodes and electrical connections between adjacent MBs were first constructed via photolithography, thermal evaporation deposition and the lift-off process. To demonstrate the versatility and high precision of photolithography technology, three configurations were designed for the interdigital current collectors, containing two, three and four fingers with different widths on one side, respectively (Fig. S2). Due to the high resolution and excellent pattern reproduction ability of photolithography, the resulting photoresist templates (Figs S3 and S4) and the final current collectors (Fig. 1b and Figs S5 and S6) had excellent consistency with the designed patterns. Subsequently, another photolithography process was carried out to create the SU-8 photoresist grooves in which the patterns were identical to the current collectors for microelectrodes and the pattern position was perfectly aligned with the current collectors (Fig. 1c and Figs S7 and S8). Then, the electrode material was ground into the slurry with appropriate fluidity and blade-coated on the patterned SU-8 photoresist surface, allowing the electrode material to fill into the photoresist grooves. To obtain intricately patterned microelectrodes, the substrate was heated on a hotplate at 100°C for ∼10 minutes. Crucially, the significant difference in the coefficients of thermal expansion between the SU-8 photoresist (−1.057 × 10^−3^°C^−1^) and the glass substrate (5.073 × 10^−6^°C^−1^) (Fig. S9) led to asynchronous length changes during heating. This thermal mismatch accumulated substantial interfacial stress, thereby reducing adhesion. As a result, the entire photoresist layer, along with the electrode material atop it, could be meticulously peeled off in one piece with the assistance of Kapton tape, leaving no residue and causing no damage to the microelectrodes and current collectors underneath, resulting in the high-yield microfabrication of high-precision microelectrode patterns on the substrate without destruction or short circuits (Fig. 1d and Fig. S10). This step is crucial for the microfabrication of thick MBs with high areal capacity and high rate capability. Besides, the binder within the electrode materials facilitated firm adhesion between the LVP and current collectors (Fig. S11). Consequently, during the photoresist peel-off, the LVP electrode materials inside the photoresist grooves tended to remain on the substrate, ultimately leaving the undamaged interdigital microelectrode pattern. It should be noted that the electrode material filled in both sides of the photoresist grooves, simultaneously serving as cathode and anode, resulting in a symmetric MB. Consequently, the electrode material employed in this strategy typically possesses multiple redox couples capable of simultaneously functioning as both cathode and anode. Besides, for integrated MESDs, electrochemical isolation between adjacent devices, which also means the isolation of electrolytes, is crucial. Therefore, the laser-cut PDMS film in a fence-like pattern was bonded with the glass substrates via O_2_ plasma treatment and heating [32–34], functioning as a barrier to confine the electrolytes. Finally, the electrolytes were precisely dripped onto the microelectrodes within the PDMS grate, completing the microfabrication of MBs.

**Figure 2.**
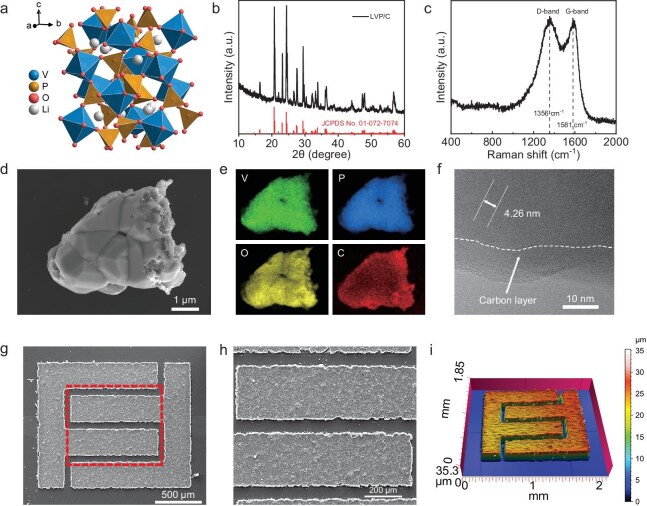
Structural and morphological characterizations of the LVP material and microelectrodes. (a) Schematic illustration of the LVP material. (b) XRD pattern, (c) Raman spectrum, (d) SEM image, (e) EDS elemental mappings and (f) high-resolution TEM image of the as-synthesized LVP material. (g) SEM image of the LVP microelectrodes. (h) Magnified SEM image of (g), showing the distinct border of the LVP microelectrodes. (i) 3D view height profile of the LVP microelectrodes.

In this work, monoclinic LVP (Fig. 2a) was selected as the electrode material to construct the symmetric MBs due to its two distinct redox couples of V^3+^/V^4+^ (3.8 V vs. Li/Li^+^ [35]) and V^3+^/V^2+^ (1.7 V vs. Li/Li^+^ [36]), which allows it to function as both cathode and anode, constructing symmetric MBs with a working voltage of ∼2.1 V. The carbon-coated LVP material was synthesized by using the sol–gel method in which the incorporated carbon-coating layer serves to enhance the electronic conductivity [37] (see details in Experimental Section in the online Supplementary file). The X-ray diffraction (XRD) pattern of the as-synthesized LVP indexed well to monoclinic LVP (JCPDS No. 01–072–7074) (Fig. 2b) and the Fourier transform infrared (FTIR) spectrum showed the characteristic bands of the PO_4_^3−^ and VO_6_ (Fig. S12). The Raman spectrum exhibited the characteristic D-band and G-band of carbon (Fig. 2c), demonstrating successful carbon coating on the LVP. A scanning electron microscopy (SEM) image revealed irregular LVP particles that were a few micrometers in size (Fig. 2d) with a uniform distribution of V, P, O and C elements, as shown by using energy dispersive spectrometer (EDS) mapping (Fig. 2e). Transmission electron microscopy (TEM) images also displayed a thin carbon layer coating the particles, with a clear boundary between the inner lattice fringe and the external amorphous carbon (Fig. 2f and Fig. S13). The SEM image of the LVP microelectrode is shown in Fig. 2g, showcasing an interdigital electrode design with a width of 300 μm and a gap of 50 μm. An electrode with even higher precision with a width of 100 μm could also be successfully fabricated (Fig. S10). The distinct border of the interdigital LVP microelectrodes and the absence of an SU8 photoresist residue could be easily noticed in the magnified SEM image (Fig. 2h), demonstrating the effectiveness of our microfabrication process. A 3D view height profile of the LVP microelectrodes (Fig. 2i) further demonstrated their structural integrity and uniform thickness from multiple dimensions. Meanwhile, the SEM image taken from an oblique view (Fig. S14) and thickness mapping (Fig. S15) clearly showed that the microelectrodes had a uniform thickness of ∼25 μm. It was indicated that the microelectrodes fabricated through our microfabrication strategy exhibit thicknesses of tens of micrometers (same as the lateral pattern resolution), significantly outperforming deposition techniques such as electrodeposition and vapor deposition.

**Figure 3.**
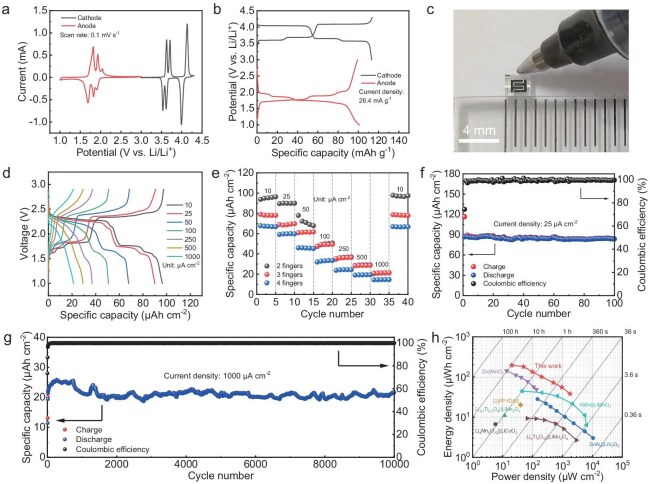
Electrochemical measurement of the LVP material and single MB. (a) CV curves and (b) GCD profiles of the LVP material at the potential range of 3.0–4.3 (cathode) and 1.0–3.0 (anode) V vs. Li/Li^+^. (c) Optical image of the MB compared with a nib. (d) GCD profiles obtained at different current densities of 2F-MB. (e) Comparison of the rate capability of 2F-MB, 3F-MB and 4F-MB. (f) Cycling performance of 2F-MB obtained at a low current density of 25 μA cm^−2^. (g) Long-term cyclability of 2F-MB. The first five cycles were activated at a small current density of 10 μA cm^−2^. (h) Ragone plot of our MB and the reported MBs [23,29,38–42] with a device area within 1 cm^2^.

To construct the symmetric MB, the electrochemical performance of the LVP material as both the cathode and the anode material was first examined. As shown in Fig. 3a, the LVP electrode exhibited two sets of redox peaks in cyclic voltammetry (CV) curves as expected, within potential ranges of 1.5– 2.1 and 3.4–4.3 V vs. Li/Li^+^, respectively. In addition, the plateaus could be found in the galvanostatic charge and discharge (GCD) profiles within the same potential range in the CV curves (Fig. 3b), demonstrating the ability of the LVP material to be simultaneously used as the cathode and anode. The GCD profiles of the LVP as cathode and anode at 0.2 C (1 C = 132 mA g^−1^) offered similar specific capacities of 113 and 99 mAh g^−1^, respectively. Given this characteristic, when constructing a symmetric MB, there is no need to adjust the ratio of the cathode to the anode; a direct 1:1 ratio can be adopted. It is manifested that the cathode and the anode can maintain the same size and thickness, indicating that using such a planar design can not only simplify the microfabrication process, but also enhance production efficiency. Besides, both the LVP cathode and anode showed decent rate performance and excellent cycling stability (Figs S16 and S17). Subsequently, the MBs, roughly the size of a pen tip, using the LVP microelectrode as the cathode and the anode, were fabricated (Fig. 3c) and a series of electrochemical measurements were carried out to study their electrochemical performance. At a current density of 10 μA cm^−2^, three pairs of major charge and discharge plateaus could be found at 1.71/1.62, 1.82/1.76 and 2.38/2.31 V in the GCD profiles (Fig. 3d), which were in good accordance with the potential difference between the LVP cathode and anode in half-cells. When the current density was increased, the two charge/discharge plateaus at the lower voltage gradually merged. To demonstrate the high precision and versatility of the peelable photoresist strategy for fabricating MBs, we prepared three types of MBs with different configurations, namely those with two, three and four fingers on one side, denoted hereafter as 2F-MB, 3F-MB and 4F-MB, respectively. Furthermore, we have systematically investigated the electrochemical performance of these three MBs (Fig. 3e and Figs S18–S20). Figure 3e shows that 2F-MB possessed the highest areal capacity of 96.4 μAh cm^−2^ based on the whole device, surpassing 3F-MB (79.0 μAh cm^−2^) and 4F-MB (67.8 μAh cm^−2^). The performance disparity of these three configurations might be related to the proportion of the active electrode area in the total area of the microdevice. As illustrated in Fig. S21 and Table S1, when the width of the fingers decreases while their numbers increase in the microdevices, the number of gaps between the fingers will increase, resulting in a reduction in the effective area ratio of the microelectrodes and, consequently, a decrease in the areal capacity. Thus, the following electrochemical measurements were all based on the 2F-MBs, owing to the highest area-utilization ratio. When the current density was increased to 1000 μA cm^−2^, a fair areal capacity of 21.2 μAh cm^−2^ could be maintained and the areal capacity could be well restored to 98.0 μAh cm^−2^ when the current density was returned to 10 μA cm^−2^ (Fig. 3e), demonstrative of decent rate capability. Further, the cycling performance of the 2F-MB was conducted. At a low current density of 25 μA cm^−2^, the capacity retention of 2F-MB after 100 cycles was 96.8% (Fig. 3f). Surprisingly, at a high current density of 1000 μA cm^−2^, the 2F-MB still demonstrated excellent cyclability with a capacity retention of 88.3% after 10 000 cycles (Fig. 3g and Fig. S22). This performance stands out as one of the best reported in the literature, in which the cycle numbers mainly did not exceed 2000 (Table S2). It was observed that the initial coulombic efficiency (ICE) of 2F-MB was very close to that of the LVP anode, in which the LVP anode may dominate the ICE and reversible capacity of the full cell (Fig. S23). The LVP anode showed excellent cycling stability during the following cycles (Fig. S17c), so the full cell still possessed an ultra-long lifespan of >10 000 cycles. To highlight the superiority of this MB, a Ragone plot containing the areal energy density and power density of the 2F-MB was given and compared with other reported MBs with the device area within 1 cm^2^ (Fig. 3h). Notably, the energy density of our 2F-MB could reach 195.5 μWh cm^−2^, surpassing those of many reported works, e.g. Li_4_Mn_5_O_12_||LiCoO_2_ (6.71 μWh cm^−2^) [38], SnN*_x_*||LiV_2_O_5_ (28.2 μWh cm^−2^) [29], Li_4/3_Ti_5/3_O_4_||LiMn_2_O_4_ (11 μWh cm^−2^) [39], Zn||MnO_2_ (141.9 μWh cm^−2^) [40], C||PPYDBS (20.2 μWh cm^−2^) [41], NiSn||LiMnO_2_ (44.9 μWh cm^−2^) [23] and Li_4_Ti_5_O_12_||LiMn_2_O_4_ (9.45 μWh cm^−2^) [42].

**Figure 4.**
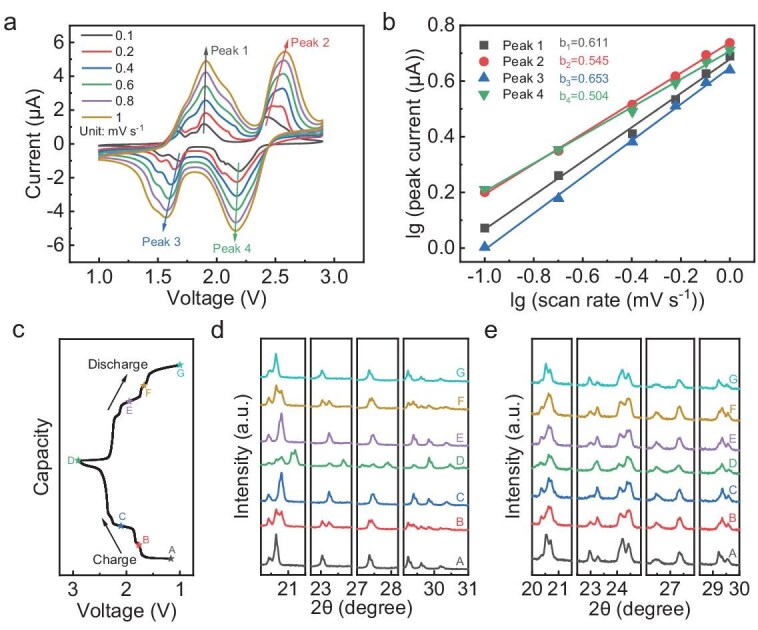
Kinetics and mechanism during the charge and discharge processes of 2F-MBs. (a) CV curves of the MB at different scan rates. (b) lg (peak current) as a function of lg (scan rate). (c) GCD profile in the second cycle of the LVP||LVP symmetric cell tested at 0.1 C (1 C = 132 mA g^−1^). *Ex situ* XRD patterns of the LVP (d) cathode and (e) anode tested at different voltages shown in (c).

CV tests at different scan rates were conducted to deeply understand the kinetics of our MBs. Upon increasing the scan rate, two pairs of redox peaks with lower voltage (1.67/1.54 and 1.89/1.69 V) would gradually merge (Fig. 4a), similarly to the GCD profiles with increasing current density (Fig. 3d). Thus, two pairs of the redox peaks remaining at the high scan rate were chosen to analyse the kinetics of the MB. The peak current (*i*_p_, μA) and the scan rate (*v*, mV s^−1^) satisfy the following equation:


\begin{eqnarray*}
{i_{\mathrm{p}}} = a{v^b},
\end{eqnarray*}

where *a* and *b* are adjustable parameters [43]. The lg (peak current) vs. lg (scan rate) plot of the four peaks (Fig. 4b) demonstrated that the *b* values were 0.611, 0.545, 0.653 and 0.504, respectively—closer to 0.5, indicating that the electrochemical reactions were dominated by the diffusion process [44]. Furthermore, to explore the reason for the excellent cycling stability of the MBs, the structure evolution of the LVP was characterized by using *ex situ* XRD patterns (Fig. 4c–e). From the GCD profiles of the LVP half-cells (Fig. 3b), the insertion and extraction of Li^+^ in the LVP cathode proceeded in three steps, in which the LVP transformed between Li_3_V_2_(PO_4_)_3_, Li_2.5_V_2_(PO_4_)_3_, Li_2_V_2_(PO_4_)_3_ and LiV_2_(PO_4_)_3_ [45]. For the LVP anode, the GCD profiles showed four charge-transfer processes (Fig. 3b) and each process involved the insertion and extraction of ∼0.5 Li^+^ [46]. Every LVP cathode and anode process showed a charge and discharge plateau in which the voltage was independent of the capacity. From Gibbs's phase rule, it could be inferred that the two-phase motion happened during the electrochemical reaction [47]. When the full cell was charging to 2.1 V, about half of the capacity was delivered. For the LVP cathode, the intensity of the diffraction peaks located at 20.65°, 23.04°, 27.36°, 29.23°, 29.62° and 30.18° decreased gradually, while the intensity of the peaks located at 20.81°, 23.24°, 29.40° and 29.84° increased, corresponding to the conversion of Li_3_V_2_(PO_4_)_3_ into Li_2_V_2_(PO_4_)_3_. When the full cell was charging to 2.9 V, the peaks located at 20.42°, 20.81°, 23.24°, 27.44° and 29.40° weakened, the peak located at 29.84° intensified and new peaks located at 21.10°, 21.20°, 23.66°, 27.15° and 27.91° emerged, corresponding to Li_2_V_2_(PO_4_)_3_ converted to LiV_2_(PO_4_)_3_ [48]. For the LVP anode, intensity changes of different peaks were also observed, in which the peaks located at 20.53°, 22.96°, 24.11° and 29.22° weakened and the peaks located at 23.25° intensified when the full cell was charging to 2.9 V, corresponding to Li_3_V_2_(PO_4_)_3_ converted to Li_5_V_2_(PO_4_)_3_ [46]. The changes in the XRD patterns confirmed the two-phase electrochemical reaction mechanism. More importantly, when the full cell was discharged, the XRD pattern of the LVP cathode and anode returned to their original state in an almost completely reversible form, during which the XRD patterns at the same voltage between charge and discharge (i.e. A–G, B–F and C–E) were almost identical. It is inferred that such excellent structure evolution reversibility of the LVP cathode and anode could enable the electrode material to have structural integrity and provide the MBs with ultra-stable cycling performance.

**Figure 5.**
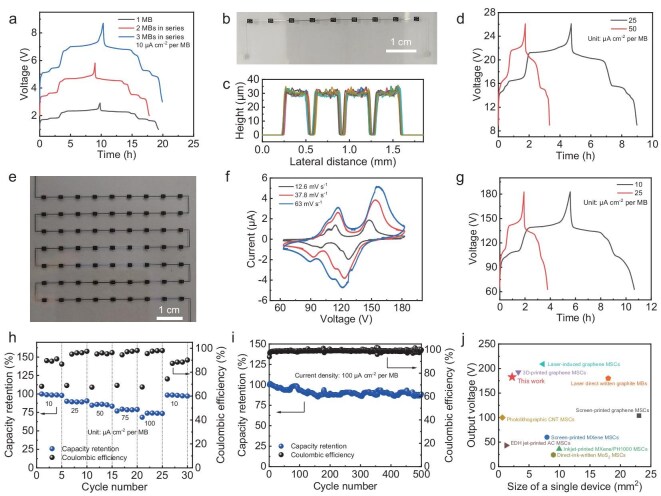
Serial integration of 2F-MBs. (a) GCD profiles of one, two and three MBs connected in series obtained at a current density of 10 μA cm^−2^ per MB. (b) Optical image of nine MBs connected in series. (c) Height profile of the microelectrodes of the nine MBs in (b). (d) GCD profiles of nine MBs connected in series at current densities of 25 and 50 μA cm^−2^ per MB. (e) Optical image of 63 MBs connected in series. (f) CV curves of 63 MBs connected in series at different scan rates. (g) GCD profiles of 63 MBs connected in series at current densities of 10 and 25 μA cm^−2^ per MB. (h) Rate performance of 63 MBs connected in series at different current densities. (i) Cycling stability of 63 MBs connected in series at a current density of 100 μA cm^−2^ per MB. (j) Output voltage comparison of our integrated MBs as a function of the single device size with reported MBs and MSCs [51–57].

It is worth noting that fabricating multiple MBs connected in series on the same substrate to achieve high voltage output is urgently required for high-voltage application scenarios, such as piezoelectric actuators [49,50]. However, to this day, developing a universal, controllable and customizable strategy for constructing monolithic integrated MBs in a highly scalable manner remains a significant challenge. To demonstrate the advantages of our strategy, we have extended this strategy to the mass production of MBs and progressively constructed integrated modules containing 2, 3, 9 and 63 MBs connected in series on the same substrate with a device spacing of 4.85 mm. Firstly, the GCD profiles of one, two and three MBs connected in series showed similar capacity and almost identical shape, and their voltage was proportional to the number of integrated MBs (Fig. 5a), demonstrating excellent performance uniformity. No capacity fading was found during the cycling test of the two MBs connected in series (Fig. S24), confirming the performance uniformity and reliability of our integrated MBs. In addition, nine MBs connected in series were also fabricated (Fig. 5b) and the thickness of the microelectrodes within the nine MBs was nearly identical (Fig. 5c), further validating the scalability of our strategy. The GCD profiles of nine MBs also showed a similar shape to one MB and the voltage was nine times that of one MB (Fig. 5d), evidencing their stable electrochemical performance. Moreover, the ultra-high output voltage integrated module with 63 MBs connected in series was successfully assembled within an area of 5.365 × 4.035 cm^2^ (Fig. 5e), which could achieve a record voltage of 182.7 V. The CV curves of this integrated MBs module (Fig. 5f) closely matched the shape of a single MB (Fig. 4a), albeit with a proportionally expanded voltage range. Similarly, the GCD profiles (Fig. 5g) also showed highly stable charge and discharge plateaus, comparable to those of the none MBs connected in series (Fig. 5d), and this integrated module could operate stably at varying current densities (Fig. 5h), confirming the superiority of our microfabrication technique. Furthermore, our integrated MBs module could stably cycle 500 times and maintain an 88.7% capacity retention (Fig. 5i), further demonstrating the advantage of our microfabrication strategy. The output voltage value that our MBs integrated module achieved is among the uppermost of the integrated MBs reported, e.g. laser direct written graphite MBs (180 V) [51], and can even compete with some MSCs, e.g. screen-printed graphene-based MSCs (104 V) [52], screen-printed MXene-based MSCs (60 V) [53], inkjet-printed MXene/PH1000-based MSCs (36 V) [54], photolithographic carbon nanotube (CNT)-based MSCs (100 V) [55], electrohydrodynamic jet-printed activated carbon-based MSCs (43.2 V) [56] and direct-ink-written MoS_2_-based MSCs (24 V) [57] (Fig. 5j and Table S3). The miniaturization of our integrated MBs (2.2275 mm^2^) also surpasses many reported works (Fig. 5j and Table S3), which could promote the rapid development of integrated MESDs in a small-scale range.

## CONCLUSION

In summary, we have demonstrated a non-destructive peeling strategy for the photolithographically patterned photoresist to realize the mass production of ultra-small, large-scale and monolithic integrated MBs for on-chip energy storage. Both intact microelectrodes and high pattern resolution could be ensured, and an ultra-small MB within 2.5 mm^2^ could be obtained. The areal capacity of the MB could reach 98.0 μAh cm^−2^ and a high energy density of 195.5 μWh cm^−2^ could be obtained, outperforming most MBs with a size of <1 cm^2^. Further, our MBs could deliver incredible cyclability, with 88.3% capacity retention after 10 000 cycles. Benefitting from the excellent scalability of photolithography and the non-destructive photoresist peeling strategy, high-voltage serial integration of our MBs could be implemented, offering an ultra-high voltage of 182.7 V, which is among the highest values reported so far. To sum up, this work offers a reliable photolithographic microfabrication strategy for the large-scale production of monolithic integrated MBs, holding great promise for the development of MESDs in the era of IoT.

## Supplementary Material

nwaf302_Supplementary_data_r.docx
