# Supplementary material for: Scalable microfabrication of monolithic integrated microbatteries with ultra-high voltage output and excellent customizability
Source: Natl Sci Rev. 2025 Jul 28;12(9):nwaf302. doi: 10.1093/nsr/nwaf302 (PMC12400807; doi:10.1093/nsr/nwaf302)
Supplement: nwaf302_Supplementary_data_r.docx [file nwaf302_supplementary_data_r.docx]

**Supplementary data**

**Scalable microfabrication of monolithic integrated microbatteries with ultrahigh voltage output and excellent customizability**

Yuan Ma^1,5^, Sen Wang^1,2,^*, Zhuobin Guo^1,5^, Xiao Wang^1^, Yuxin Ma^1,5^, Yinghua Fu^1,5^, Hanqing Liu^1,5^, Shengwei Li^1^, Yao Lu^3,^*, Zhizhang Yuan^4^ and Zhong-Shuai Wu^1,^*

^1^State Key Laboratory of Catalysis, Dalian Institute of Chemical Physics, Chinese Academy of Sciences, Dalian 116023, China;

^2^School of Transportation Engineering, Dalian Jiaotong University, Dalian 116028, China;

^3^State Key Laboratory of Phytochemistry and Natural Medicines, Dalian Institute of Chemical Physics, Chinese Academy of Sciences, Dalian 116023, China;

^4^Division of Energy Storage, Dalian National Laboratory for Clean Energy, Dalian Institute of Chemical Physics, Chinese Academy of Sciences, Dalian 116023, China;

^5^University of Chinese Academy of Sciences, Beijing 100049, China

***Corresponding authors**. E-mails: senwang@dicp.ac.cn; luyao@dicp.ac.cn; wuzs@dicp.ac.cn

**Experimental Section**

**Synthesis of the LVP material:** LVP was prepared using the sol-gel method[1]. Specifically, citric acid monohydrate (Sinopharm, 99.5%, 10 mmol), NH_4_VO_3_ (Sinopharm, 99%, 20 mmol), NH_4_H_2_PO_4_ (Aladdin, 99%, 30 mmol), and LiOH·H_2_O (Aladdin, 99%, 30 mmol) were dissolved in deionized (DI) water (200 mL) and magnetic stirred in a water bath at 60℃ to attain a chartreuse solution. After the mixture became a black-green gel, it was dried at 80℃ for 12 h in the vacuum oven. The dried solids were ground into powder, pelletized at 150 bar, and pre-calcined in a tube furnace at 300℃ for 4 h under the Ar atmosphere with a heating rate of 5℃ min^−1^, yielding the brown solids. The brown solids were ground into powder, pelletized at 150 bar, and calcined at 800℃ in the Ar atmosphere for 8 h at a heating rate of 5℃ min^−1^ in a tube furnace to obtain the black solids. After grinding the black solids into powder, the LVP material was obtained.

**Fabrication of LiTFSI/P_14_TFSI electrolyte:** LiTFSI (DoDoChem, 99.9%, 10 mmol) was dissolved into P_14_TFSI (Lanzhou Greenchem ILs, 99%, 10 mL) ionic liquid in the Ar atmosphere glove box with H_2_O and O_2_ less than 0.01 ppm. The mixture was transferred from the glove box and heated at 120℃ for 24 h in the vacuum oven. Afterward, the electrolyte was obtained and stored in the Ar atmosphere glove box[2].

**Fabrication of current collectors and LVP microelectrodes arrays:** The AZ P4620 photoresist (Merck) was first spin-coated onto the O_2_ plasma-cleaned glass substrate at 500 rpm for 18 s and 1500 rpm for 60 s. The sample was then soft baked at 110℃ for 80 s for solvent removal and further subjected to 280 mJ cm^−2^ of ultraviolet exposure at 365 nm (URE-2000/35 mask aligner, Institute of Optics and Electronics, CAS) through a film photomask (Kunshan Kaisheng Electronics Co., Ltd). Then, the sample was developed using 2.38 wt% tetramethylammonium hydroxide (aq) (JHM Electronic Materials) at 150 rpm for 3.5 min and rinsed with DI water to get the photoresist pattern. Afterward, 20 nm Ti and 250 nm Al were thermally evaporated and deposited (Fujian Zijin Innovation Applied Materials Co., Ltd) on the photoresist-patterned glass substrate. A typical lift-off process enabled by immersing the Ti/Al deposited sample into ethanol was conducted to obtain the interdigital current collectors. For patterning the microelectrodes, the SU-8 3035 photoresist (Kayaku Advanced Materials) was spin-coated onto the Ti/Al-deposited glass substrate at 500 rpm for 18 s and 4000 rpm for 30 s. The samples were then soft baked at 95℃ for 20 min for solvent removal and subjected to 100 mJ cm^−2^ of ultraviolet exposure at 365 nm through a film photomask. The exposed samples were then post-exposure baked at 65℃ for 1 min and 95℃ for 5 min. Subsequently, the samples were developed using ethyl lactate (Damao, 99%) at 120 rpm for 5 min to obtain the photoresist grooves. The substrates with photoresist grooves were subjected to O_2_ plasma treatment to enhance the hydrophilia. The LVP material, Super P conductive additive (Canrd), and carboxymethylcellulose sodium (CMC) (Sinopharm, 800~1200 mPa s^−1^) binder with a ratio of 8:1.3:0.7 in weight were ground together with DI water, and the obtained electrode slurry was blade-coated into the photoresist grooves. Until the electrode slurry dries naturally, Kapton tape was applied to the samples and heated at 100℃ on a hot plate. During heating, the photoresist will soften, and the whole photoresist film, together with the Kapton tape, was carefully peeled off, leaving the interdigital microelectrodes on the substrate. The microelectrodes were heated at 105℃ overnight in a vacuum oven for further drying.

**Addition of the electrolyte:** The PDMS film (Hangzhou Westru Technology Co., Ltd.) was first cut by laser (Shenzhen Laser Source Technology Co., Ltd) with small square holes. Then, the PDMS with the square holes and the glass substrate with microelectrodes were treated in O_2_ plasma for 1 min. After O_2_ plasma treatment, the treated surface of the PDMS and glass substrate were carefully aligned to ensure the LVP microelectrodes were within the square holes and fit tightly. The irreversible bond between PDMS and glass substrate was enabled by heating at 80℃ for 5 min, and the PDMS grate was obtained. The electrolyte was dripped onto the microelectrodes in the Ar atmosphere glove box.

**Materials characterization:** The structures were investigated by XRD pattern (Rigaku SmartLab), Raman spectroscopy (HORIBA JOBIN YVON LabRAM HR800), FTIR spectroscopy (Thermo Scientific Nicolet iS50), thermogravimetric (TG) analysis (PerkinElmer Pyris Diamond TG/DTA), dynamic thermal mechanical analysis (DMA) (TA Q800) and thermal mechanical analysis (TMA) (TA TMA Q400). The morphologies were characterized by SEM (FEI Quanta 200F, JEOL JSM-7900F) and TEM (HITACHI HT7700, FEI Tecnai G2 F30). The element composition was analyzed by EDS detectors (Oxford X-Max^N^ 80, Oxford Ultim Extreme). The optical micrographs were recorded by optical microscope (Olympus BX53MRF-S). The thickness test of the photoresist and microelectrodes was investigated by the KLA Tencor D-600 stylus profiler.

**Electrochemical measurement:** The CV and GCD test of single MB and the GCD test of 2 MBs connected in series were conducted by the LANHE M340A precision battery tester. The GCD profiles of 3, 9, and 63 MBs connected in series and the CV curves of 63 MBs connected in series were conducted by Keithley 2450 SourceMeter. All the electrochemical measurements of MBs were carried out in the Ar glove box. The half-cells of the LVP were assembled into coin cells (CR2016) in the Ar glove box using 2500 PP film as separator and Li foil as counter and reference electrode. The working electrode (12 mm in diameter) for half-cells consisted of LVP material, Super P additive, and CMC binder (8:1:1 in weight). The electrolyte for half-cells consisted of 1 M LiPF_6_ in dimethyl carbonate (DMC): ethylene carbonate (EC): ethyl methyl carbonate (EMC) = 1:1:1 vol% with 1% vinylene carbonate (VC) (DoDoChem). The ex-situ XRD characterizations were based on the coin cells (CR2016) containing the LVP cathode (12 mm in diameter), 2500 PP separator, LVP anode (13 mm in diameter), and 1 M LiTFSI/P_14_TFSI electrolyte. The GCD profiles of the half-cells and coin-type full cells were conducted by the LANHE CT3001A battery tester. The CV curves of the half-cells were conducted by the CHI760E electrochemical workstation.

**Calculation:** The capacity *Q* (μAh) and energy *E* (μWh) of the MBs were directly recorded during the GCD test through the equations (1) and (2), respectively:

$$Q=\int_{0}^{t_{0}} Idt (1)$$

$$E=\int_{0}^{t_{0}} UIdt (2)$$

where *t*_0_ (h) is the charge or discharge time in one cycle, *I* (μA) is the current, and *U* (V) is the voltage of the batteries.

The areal capacity *Q*_areal_ (μAh cm^−2^) and areal energy density *E*_areal_ (μWh cm^−2^) were calculated following the equations (3) and (4), respectively[3]:

$$Q_{\mathrm{areal}}=\frac{Q}{S} (3)$$

$$E_{\mathrm{areal}}=\frac{E}{S} (4)$$

where *S* (cm^2^) is the area of the MB, including the microelectrodes and the gap between electrodes.

The areal power density *P*_areal_ (μW cm^−2^) was calculated following the equation (5):

$$P_{\mathrm{areal}}=\frac{E_{\mathrm{areal}}}{t_{0}} (5)$$

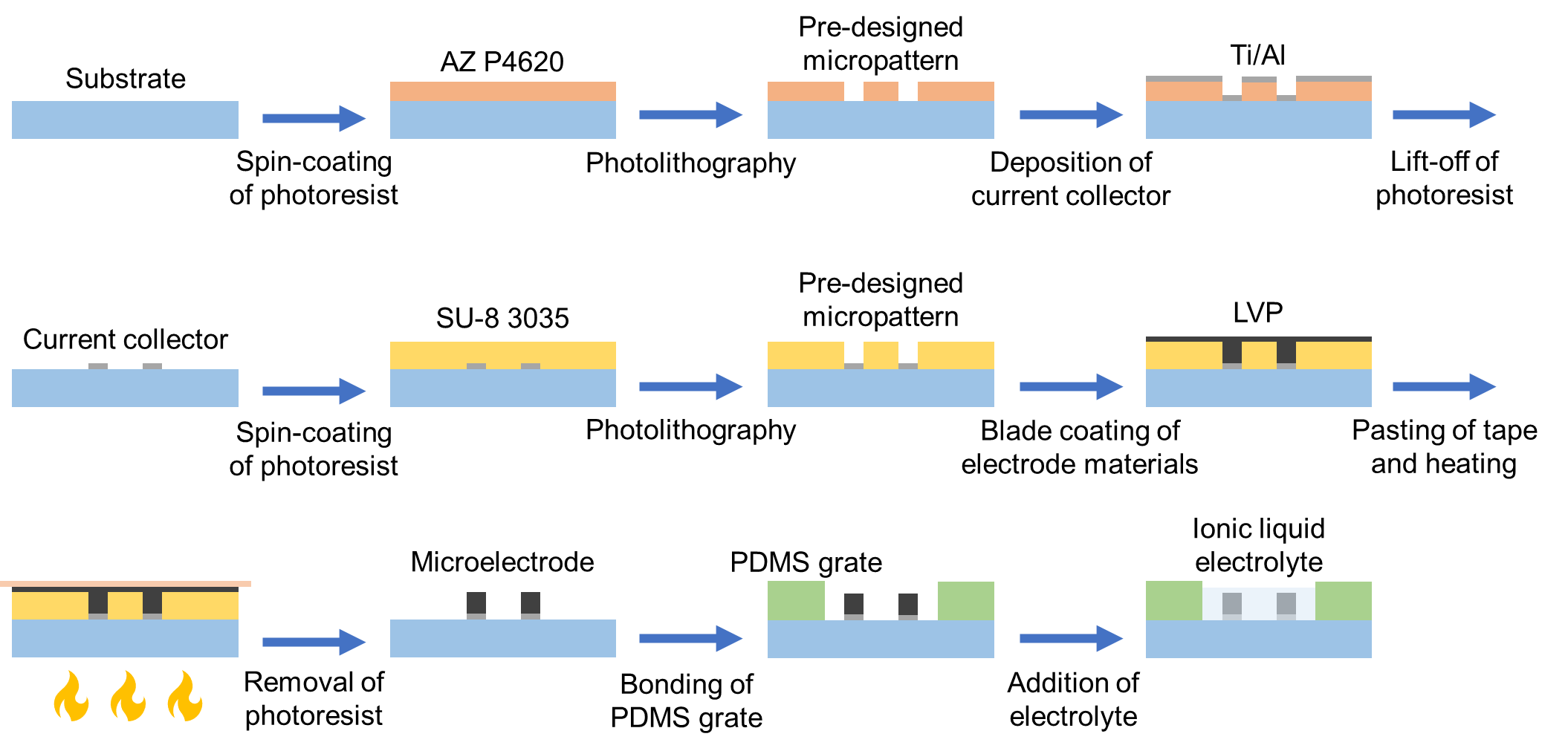


**Figure S1.** Schematic illustration of the detailed microfabrication process of MBs.


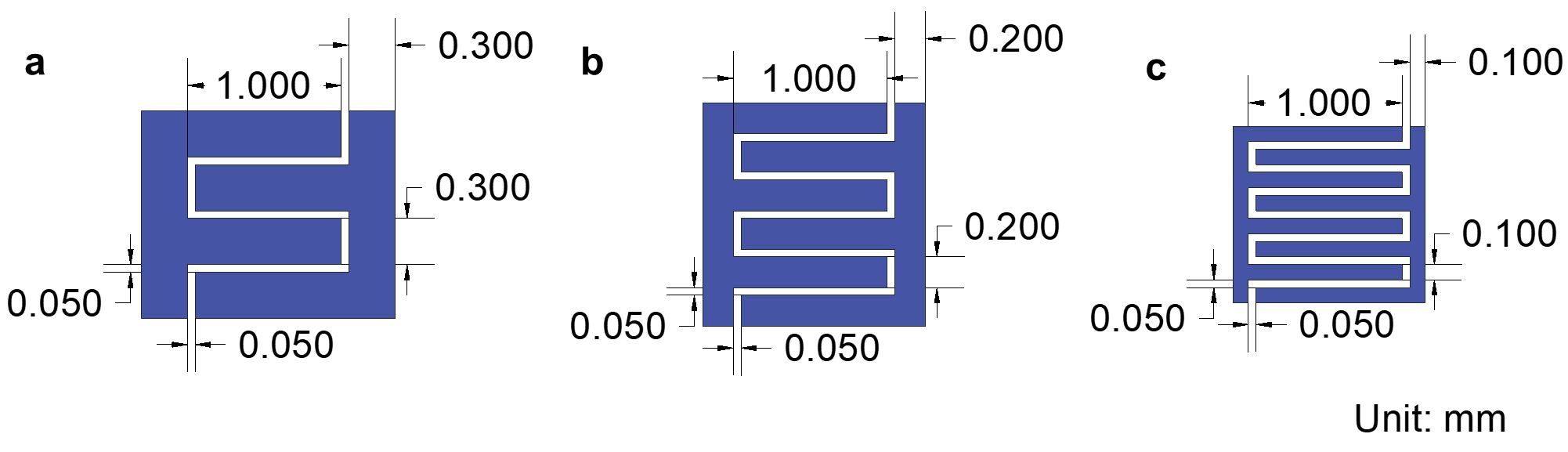


**Figure S2.** Schematic illustration of size parameters of (a) 2F-MB, (b) 3F-MB, and (c) 4F-MB. For all three configurations, the electrode length is 1 mm, and the gap between the cathode and anode is 0.05 mm. The electrode width is 0.3, 0.2, and 0.1 mm for 2F-MB, 3F-MB, and 4F-MB, respectively.


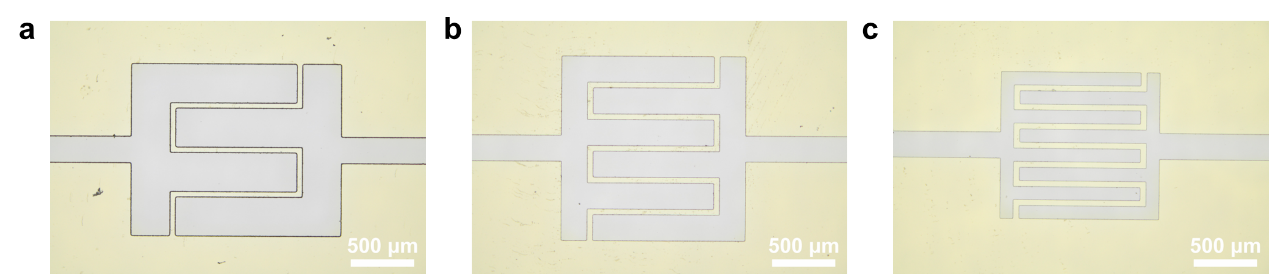


**Figure S3.** Optical microscope images of AZ P4620 photoresist template used to define the pattern of current collectors in (a) 2F-MB, (b) 3F-MB, and (c) 4F-MB.


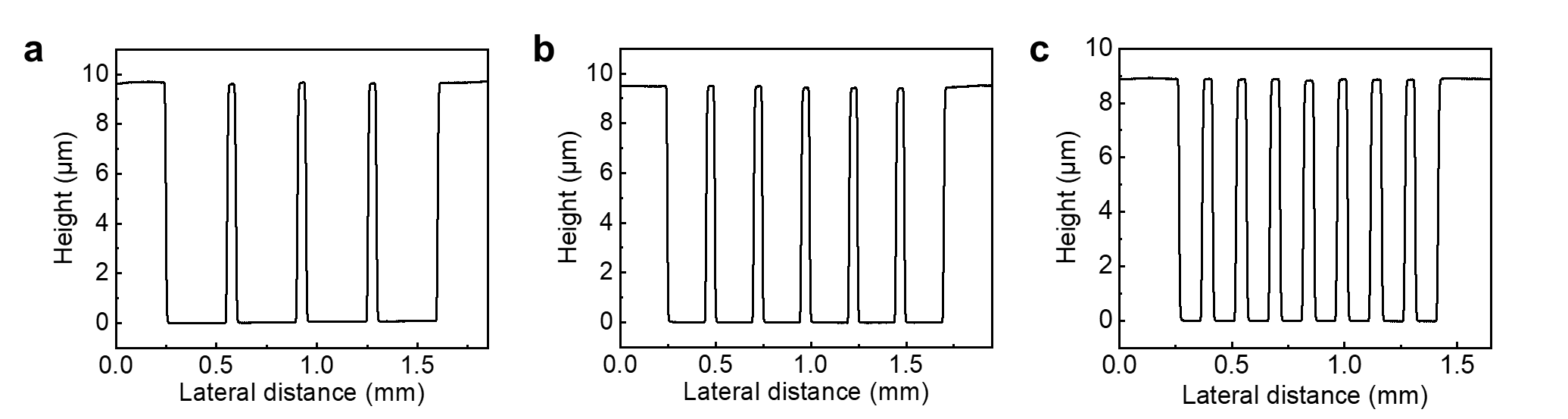


**Figure S4.** Height profiles of AZ P4620 photoresist template used to define the micropatterns of current collectors in (a) 2F-MB, (b) 3F-MB, and (c) 4F-MB.


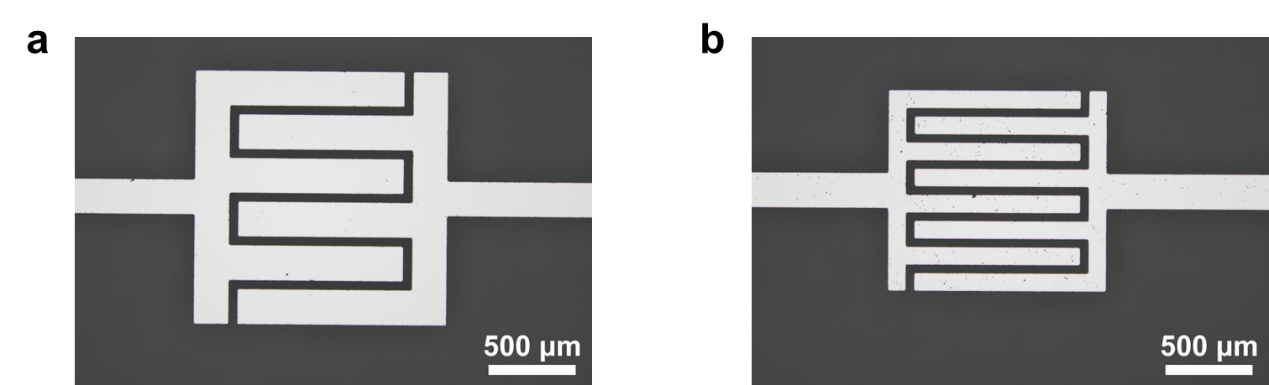


**Figure S5.** Optical microscope images of current collectors in (a) 3F-MB and (b) 4F-MB.


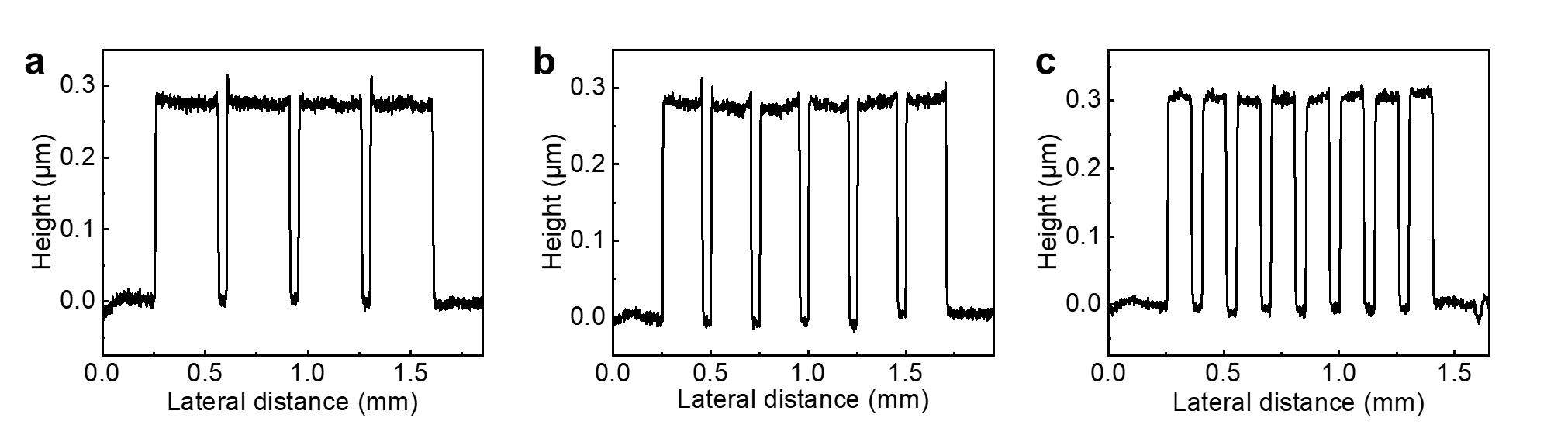


**Figure S6.** Height profile of current collectors in (a) 2F-MB, (b) 3F-MB, and (c) 4F-MB.


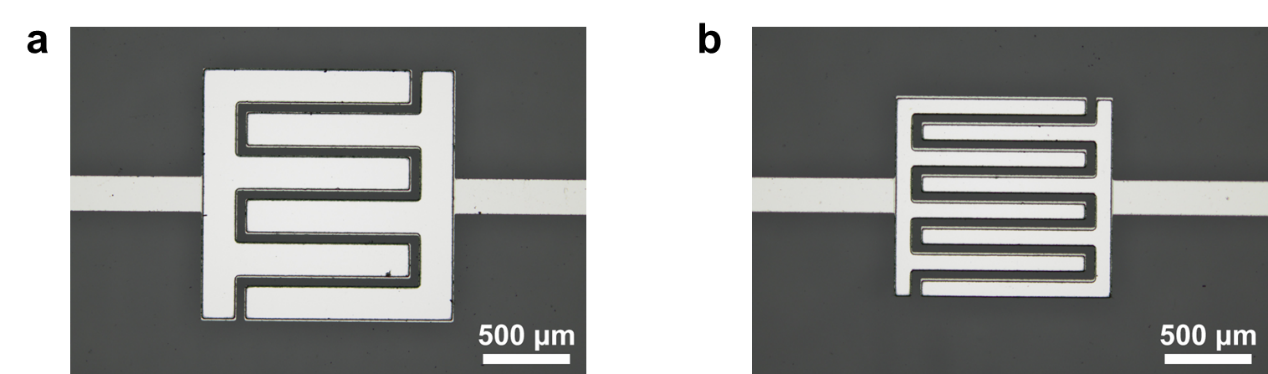


**Figure S7.** Optical microscope images of SU-8 3035 photoresist template used to define the pattern of microelectrodes in (a) 3F-MB and (b) 4F-MB.


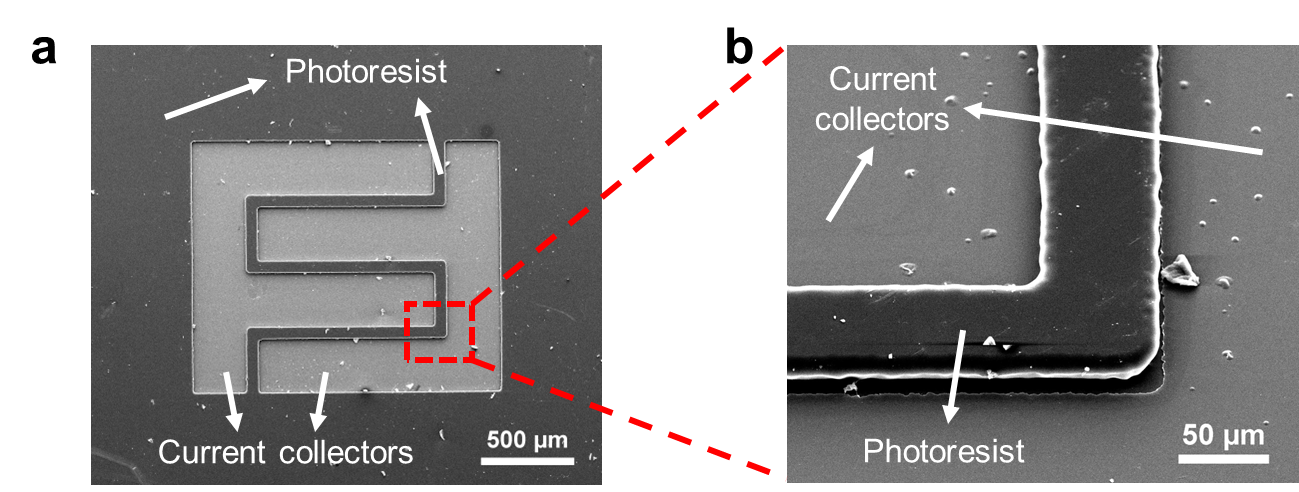


**Figure S8.** SEM images of SU-8 3035 photoresist template. (a) Top view SEM image of SU-8 3035 photoresist template used to define the pattern of microelectrodes. (b) Tilt view SEM image of SU-8 3035 photoresist template corresponding to the magnified area marked by the red dashed rectangle in (a).


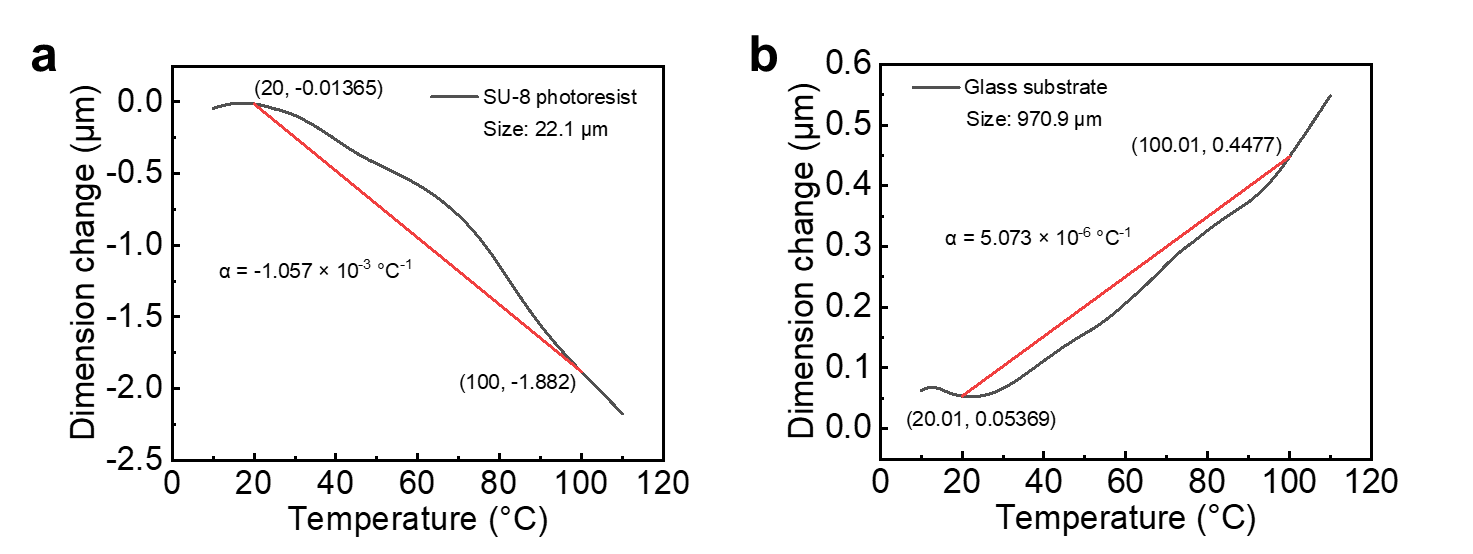


**Figure S9.** Thermal analysis of the patterned SU-8 3035 photoresist and glass substrate. (a) TMA curve of the patterned SU-8 photoresist. (b) TMA curve of the glass substrate.


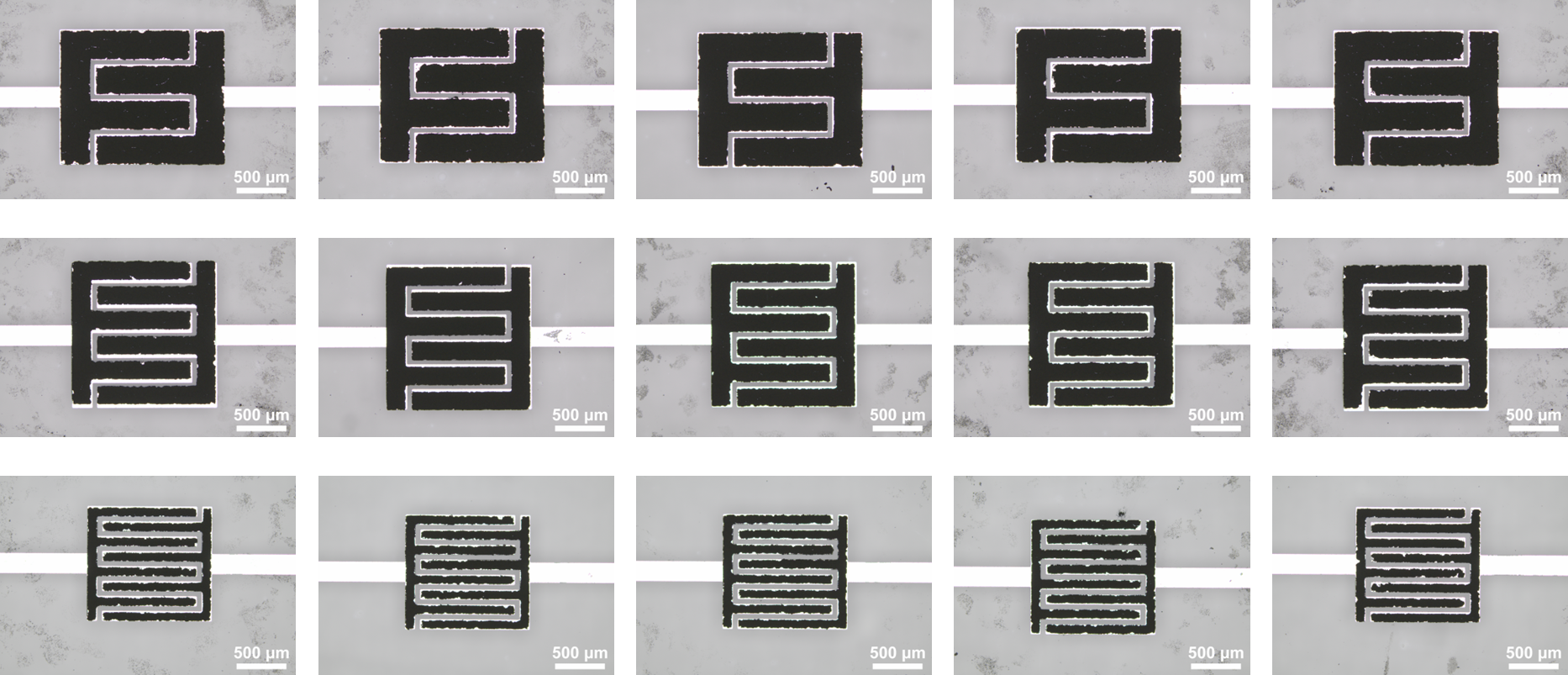


**Figure S10.** Optical images of microelectrodes in 2F-MB (top), 3F-MB (middle), and 4F-MB (bottom), respectively.


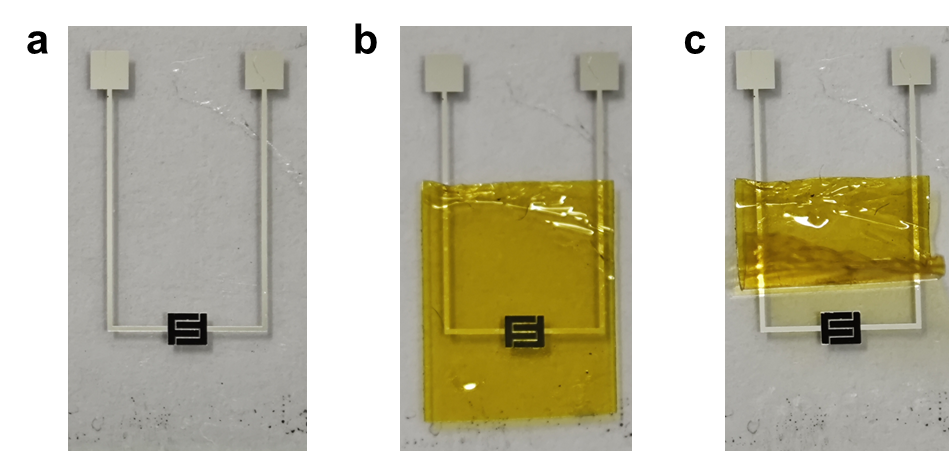


**Figure S11.** Optical images of the microelectrode at different states. (a) Optical image of the as-prepared microelectrode before being pasted with Kapton tape. (b) Optical image of the microelectrode after being pasted with Kapton tape. (c) Optical image of the microelectrode after the Kapton tape was peeled off.

It was demonstrated that even when Kapton tape was pasted onto the as-prepared microelectrode and then peeled off, the microelectrode still remained intact on the substrate, and its pattern was unaffected, demonstrating the firm adhesion between the electrode materials and substrate.


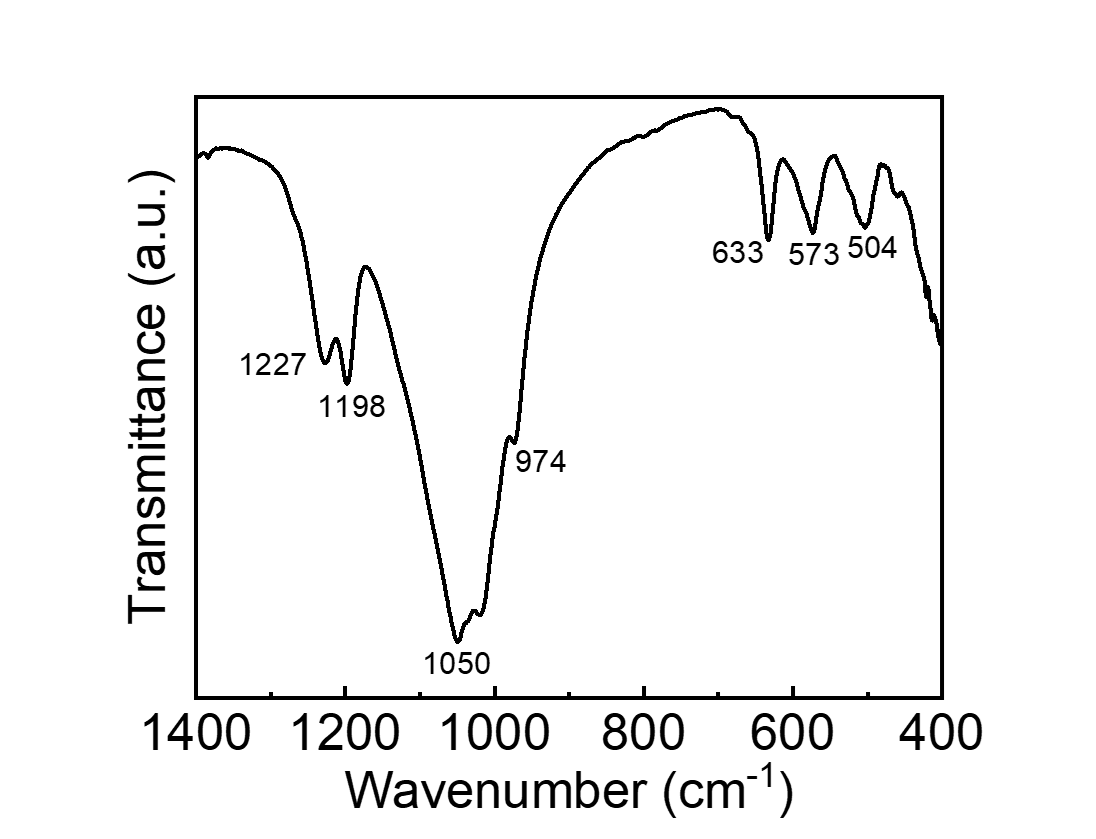


**Figure S12.** FTIR spectrum of the LVP material. The peaks at 1227 and 1198 cm^−1^ can be assigned to the stretching vibration of the terminal PO_4_^3−^. The peaks at 1050 and 573 cm^−1^ show the *ν*_3_ and *ν*_4_ modes of PO_4_^3−^, respectively. The peaks at 974, 633, and 504 cm^−1^ reveal the *ν*_2_, *ν*_1_, and *ν*_4_ modes of V^3+^ in VO_6_, respectively[4-6]. It is verified that the structure comprises PO_4_^3−^ and VO_6_, and V valence can be determined as +3.


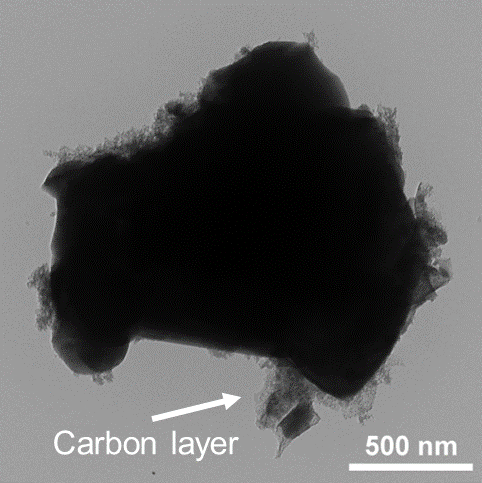


**Figure S13.** TEM image of the as-synthesized LVP material.


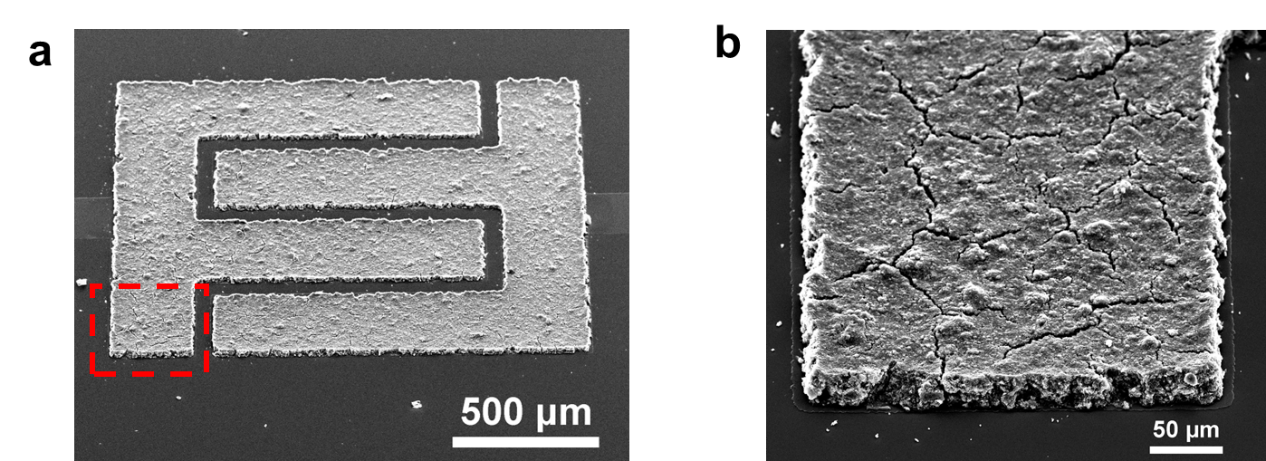


**Figure S14.** SEM images of the LVP microelectrode. (a) Tilt view SEM image of the LVP microelectrode. (b) Magnified SEM image marked by the red dashed rectangle in (a).


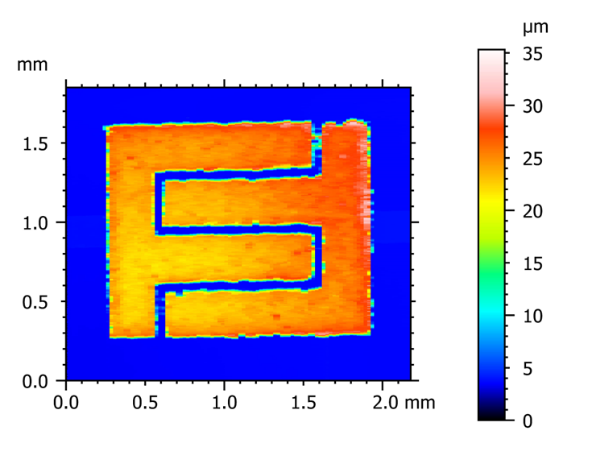


**Figure S15.** Pseudo-color view of the LVP microelectrode.


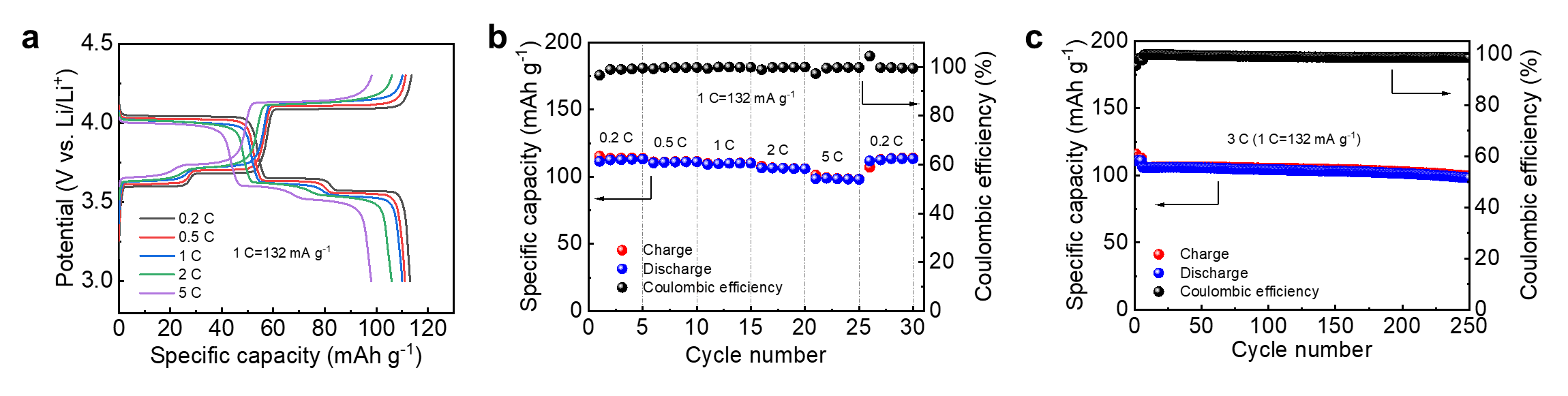


**Figure S16.** Electrochemical performance of the LVP cathode. (a) GCD profiles of LVP cathode at different current densities. (b) Rate performance of LVP cathode at different current densities. (c) Cycling performance of LVP cathode.


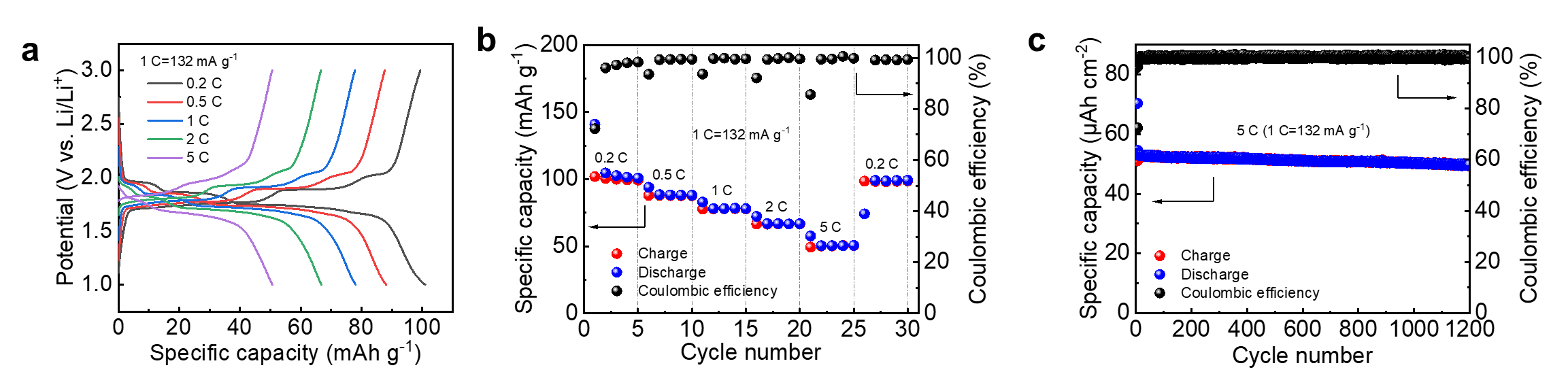


**Figure S17.** Electrochemical performance of the LVP anode. (a) GCD profiles of LVP anode at different current densities. (b) Rate performance of LVP anode at different current densities. (c) Cycling performance of LVP anode.


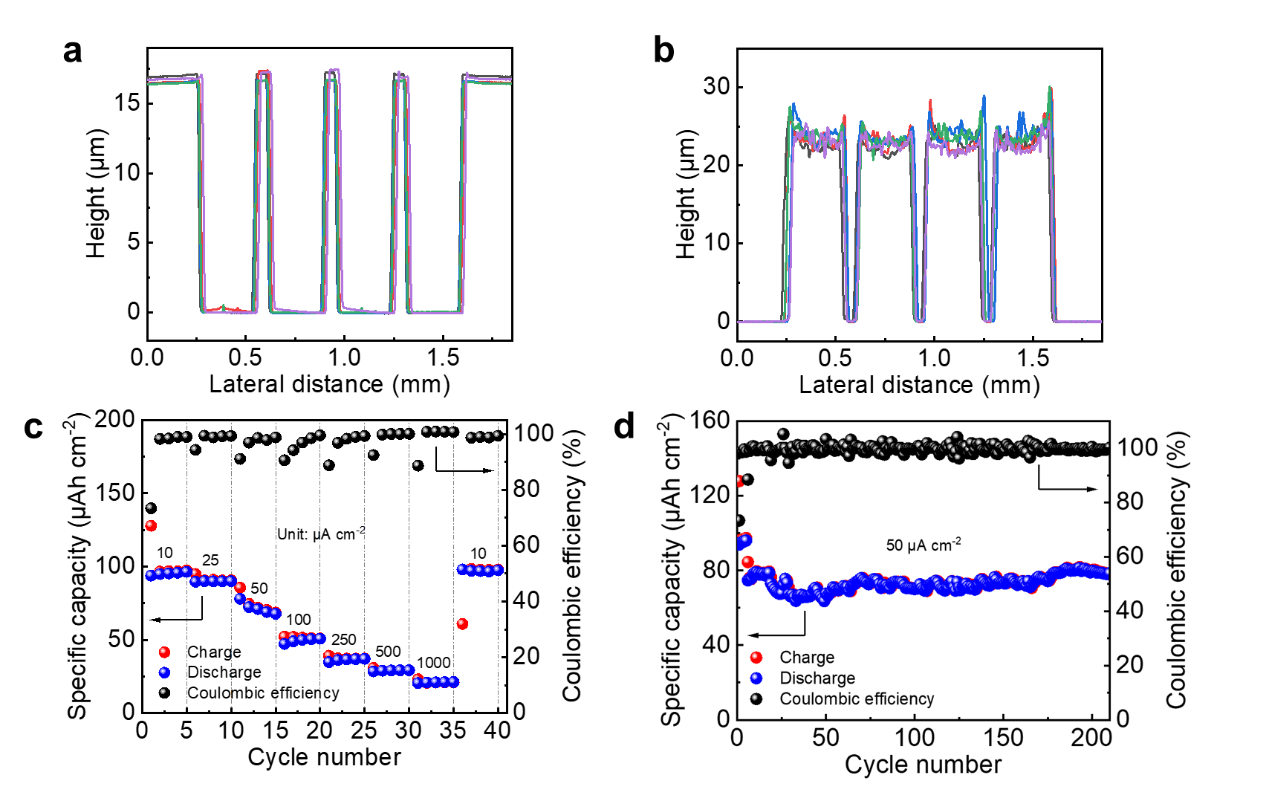


**Figure S18.** Height profile and electrochemical test of 2F-MBs. (a) Height profile of five SU-8 3035 photoresist templates used to define the pattern of microelectrodes in 2F-MB. (b) Height profile of five LVP microelectrodes in 2F-MB. (c) Rate capability of 2F-MB obtained at different current densities. (d) Cycling stability of 2F-MB at the current density of 50 μA cm^−2^.


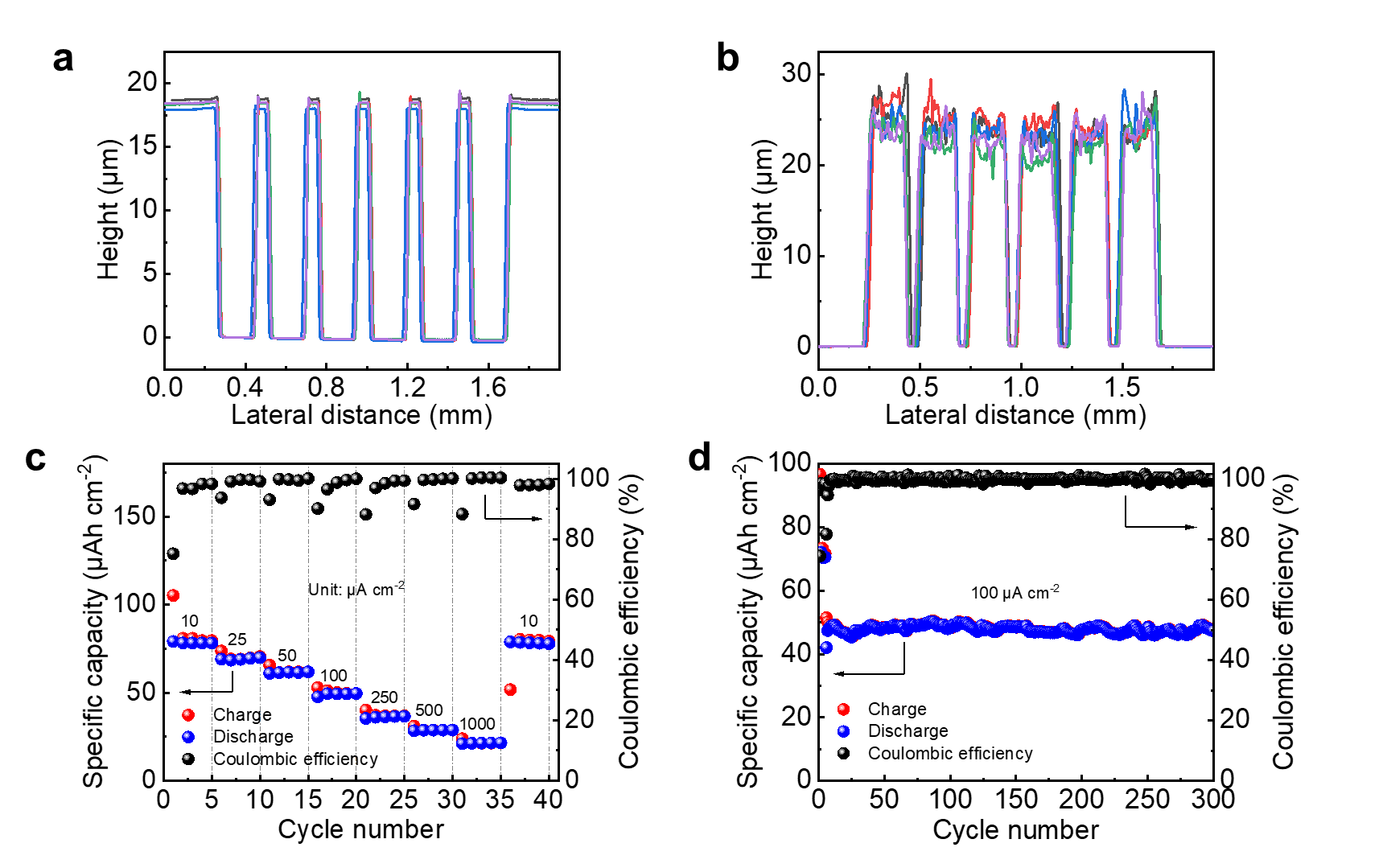


**Figure S19.** Height profile and electrochemical test of 3F-MBs. (a) Height profile of five SU-8 3035 photoresist templates used to define the pattern of microelectrodes in 3F-MB. (b) Height profile of five LVP microelectrodes in 3F-MB. (c) Rate performance of 3F-MB at different current densities. (d) Cyclability of 3F-MB at the current density of 100 μA cm^−2^.


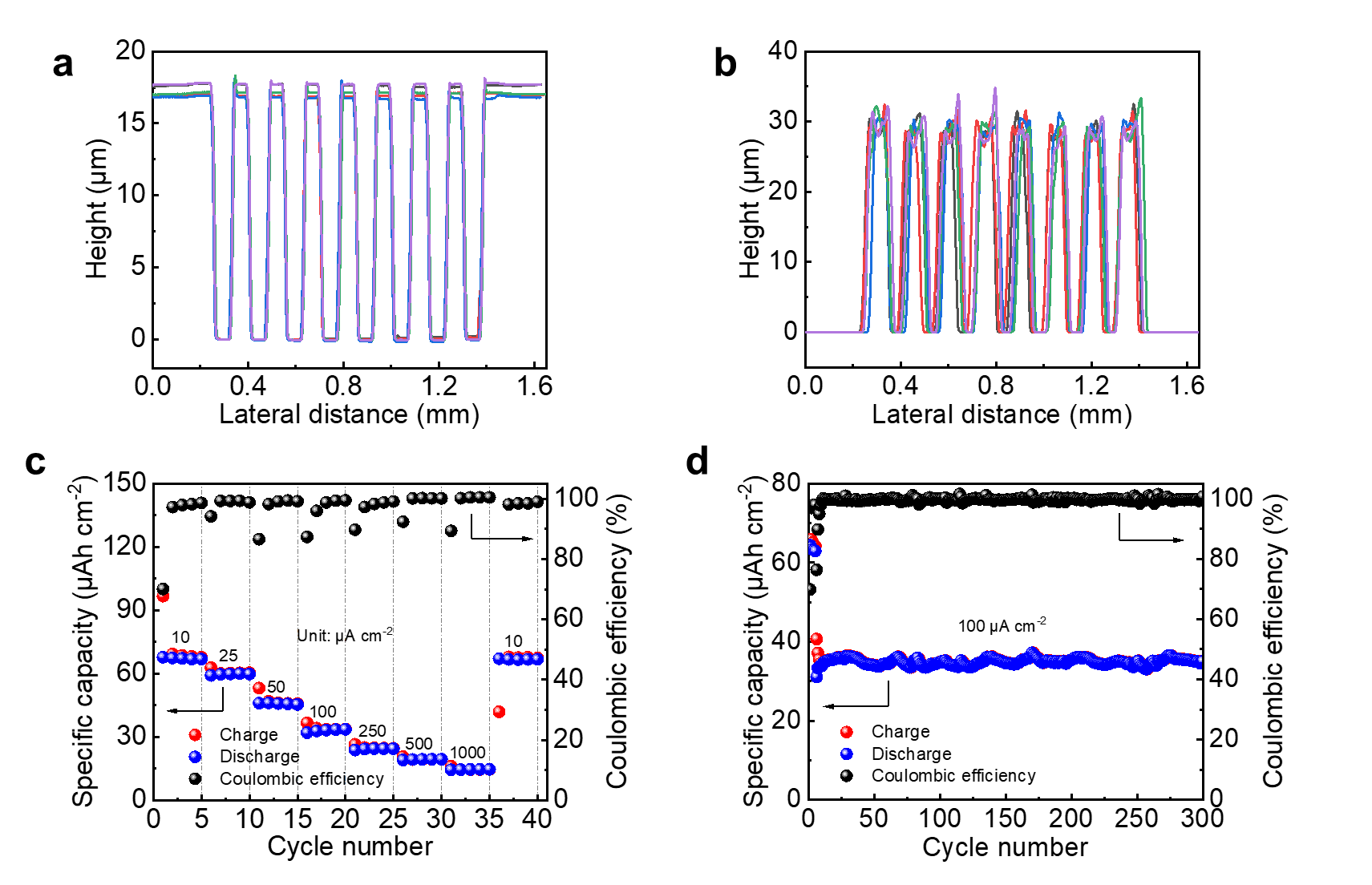


**Figure S20.** Height profile and electrochemical test of 4F-MBs. (a) Height profile of five SU-8 3035 photoresist templates used to define the pattern of microelectrodes in 4F-MB. (b) Height profile of five LVP microelectrodes in 4F-MB. (c) Rate performance of 4F-MB at different current densities. (d) Cycling stability of 4F-MB at the current density of 100 μA cm^−2^.


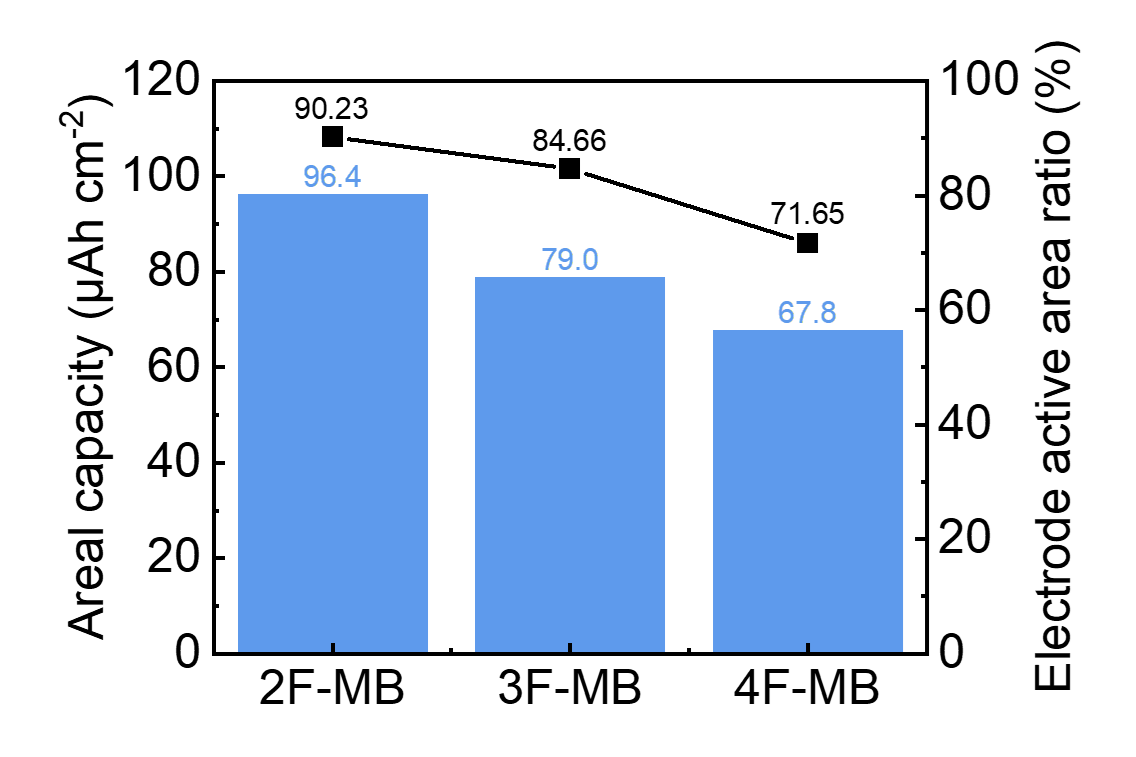


**Figure S21.** Comparison of the areal capacity and the electrode active area ratio of 2F-MB, 3F-MB, and 4F-MB.


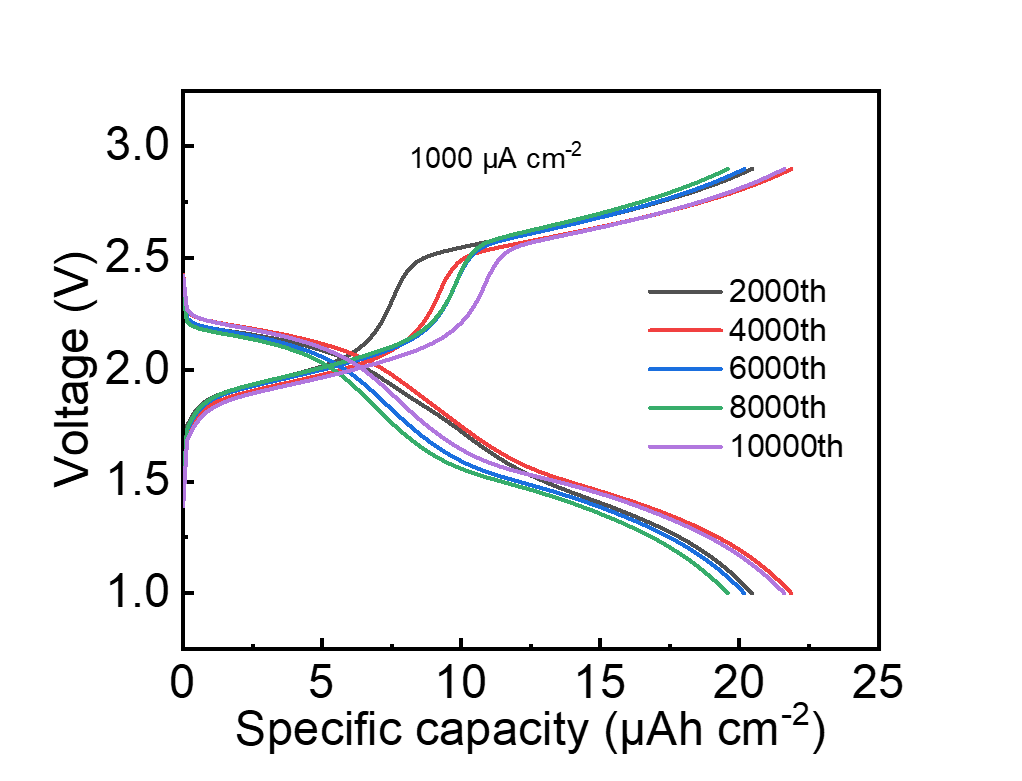


**Figure S22.** GCD profiles of the 2F-MB at different cycles during the long-term cycling test.


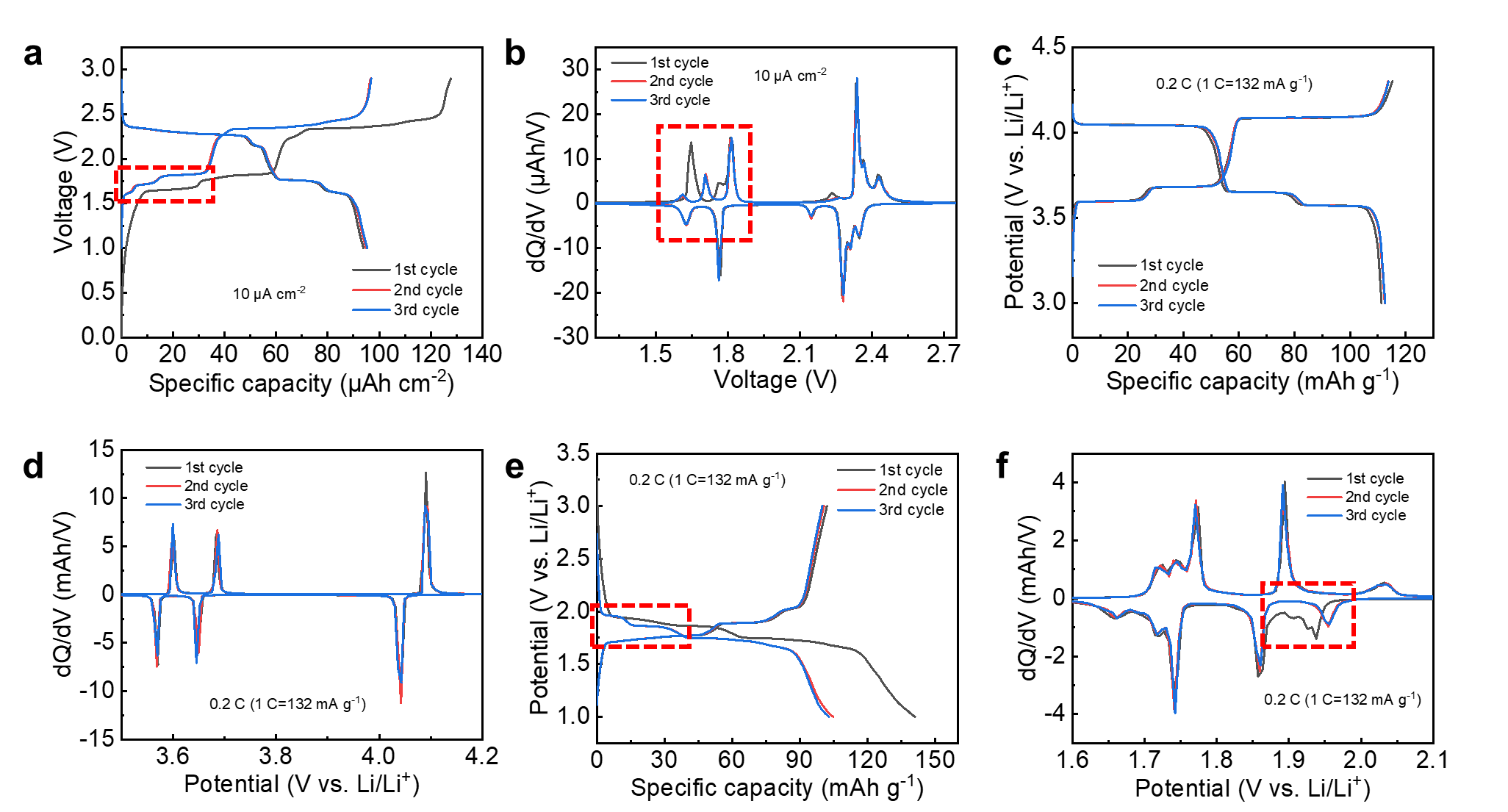


**Figure S23.** GCD and d*Q*/d*V* profiles of 2F-MB, LVP cathode, and LVP anode. (a) The first three cycles of GCD profiles of 2F-MB. (b) The first three cycles d*Q*/d*V* profiles of 2F-MB. (c) The first three cycles of GCD profiles of LVP cathode. (d) The first three cycles d*Q*/d*V* profiles of LVP cathode. (e) The first three cycles of GCD profiles of LVP anode. (f) The first three cycles d*Q*/d*V* profiles of LVP anode.

The ICE of the 2F-MB, LVP cathode, and LVP anode was 73.42%, 96.60%, and 72.30%, respectively. Figures S23c and d showed nearly no difference in the first three GCD profiles of the LVP cathode. However, the first-cycle GCD profile of the LVP anode differed notably from the second and third cycles: a relatively long plateau emerged at ~1.94 V vs. Li/Li^+^ during the first cycle (Figure S23e), corresponding to a broad peak in the d*Q*/d*V* profile (Figure S23f), while shorter plateaus at slightly higher potentials (~1.96 V vs. Li/Li^+^) appeared in subsequent cycles, corresponding to the sharp d*Q*/d*V* peaks. These cycle-dependent differences (marked by red dashed rectangles) indicated an irreversible process in the first cycle, causing low ICE. For GCD profiles of the full cell (Figure S23a and b), 2F-MB exhibited a long ~1.65 V plateau during the first charge, which shortened and shifted to ~1.62 V in subsequent cycles (highlighted by red dashed rectangles). It is indicated that the variation trend in the GCD profiles of the full cell was similar to that of the LVP anode, in which the LVP anode may dominate the ICE of the full cell.


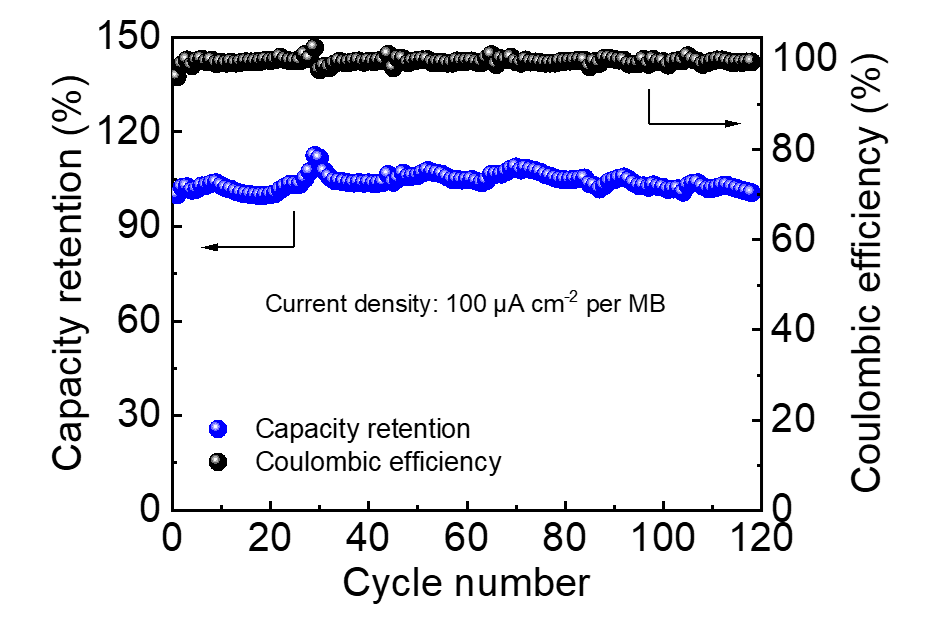


**Figure S24.** Cycling performance of two MBs connected in series.

**Table S1.** Comparison of the areal capacity, the device area, the electrode area, and the ratio of the electrode area and device area in different configurations

| **Configuration** | **Device area (mm^2^)** | **Electrode area (mm^2^)** | **Ratio of the electrode area and device area (%)** |
| --- | --- | --- | --- |
| 2F-MB | 2.2275 | 2.01 | 90.23 |
| 3F-MB | 2.1025 | 1.78 | 84.66 |
| 4F-MB | 1.4375 | 1.03 | 71.65 |

**Table S2**. Performance comparison of our single MB with the reported works

| **MB** | **Areal capacity/rate performance**  **(μAh cm****^−2^)** | **Cycling stability** | **Maximum areal energy density (μWh cm^−2^)**  **Maximum power density (μW cm^−2^)** | **References** |
| --- | --- | --- | --- | --- |
| Li_3_V_2_(PO_4_)_3_\|\|Li_3_V_2_(PO_4_)_3_ | 98.0 (10 μA cm^−2^)  21.2 (1000 μA cm^−2^) | 10 000 cycles, 88.3% (1000 μA cm^−2^) | 195.5  1801.1 | This work |
| Li_4_Mn_5_O_12_\|\|LiCoO_2_ | 7.3 (6.25 μA cm^−2^)  – | 3 cycles, 87.3% (6.25 μA cm^−2^) | 6.71  5.74 | [7] |
| SnN_x_\|\|LiV_2_O_5_ | 29.2 (100 μA cm^−2^)  2.8 (10 000 μA cm^−2^) | 100 cycles, 63.5% (100 μA cm^−2^) | 28.2  10 521.3 | [8] |
| Zn\|\|MnO_x_/PPy | 110 (0.2 mA cm^−2^)  38.5 (1 mA cm^−2^) | 200 cycles, no attenuation | –  – | [9] |
| Zn\|\|VO_2_(B)-MWCNTs | 314.7 (0.14 mA cm^−2^)  163.7 (1.43 mA cm^−2^) | 200 cycles, 71.8% (3.14 mA cm^−2^) | 188.8  610 | [10] |
| Zn\|\|MnO_2_ | 13 (20 C)  7 (800 C) | 450 cycles, no attenuation (50 C) | 16.3  11 090 | [11] |
| Zn\|\|NiCo LDH@CC | 92 (0.8 mA cm^−2^)  67 (3 mA cm^−2^) | 300 cycles, 91.2% (3 mA cm^−2^) | 146  4479 | [12] |
| Zn\|\|Ni-Ni(OH)_2_ | 150.1 (1 mA cm^−2^)  88.9 (20 mA cm^−2^) | 1800 cycles, 74.6% (10 mA cm^−2^) | 260  33 800 | [13] |
| Li\|\|V_2_O_5_ | 150 (1 C)  50 (100 C) | 550 cycles, 73% (1 C, Ar)  200 cycles, 77% (1 C, Air) | 345  75 500 | [14] |
| Zn@CNT\|\|NH_4_CuHCF-P | 220 (3 mA cm^−2^)  200 (50 mA cm^−2^) | 1000 cycles, 94% (20 mA cm^−2^) | 396  83 850 | [15] |
| Zn\|\|Ag | 220 (3 mA cm^−2^)  200 (7 mA cm^−2^) | 10 cycles, 95.9% (5 mA cm^−2^) | 320  – | [16] |
| Zn\|\|MnO_2_ | 300 (200 μA cm^−2^)  130 (500 μA cm^−2^) | 300 cycles, 34.8% (500 μA cm^−2^) | 458  – | [17] |
| Li_4/3_Ti_5/3_O_4_\|\|LiMn_2_O_4_ | 4.5 (1 C)  2.4 (50 C) | – | 11  – | [18] |
| Li_4_Ti_5_O_12_\|\|LiCoO_2_ | 270 (2 C)  170 (20 C) | 5 cycles, 94.7% (2 C) | –  – | [19] |
| Li\|\|LiCoO_2_ | 146 (130 μA cm^−2^)  120 (235 μA cm^−2^) | 120 cycles, 70% (130 μA cm^−2^) | –  – | [20] |
| InGaZnO\|\|V_2_O_5_ | 9.8 (1 μA cm^−2^)  4.8 (14 μA cm^−2^) | 300 cycles, no attenuation (14 μA cm^−2^) | –  – | [21] |
| Zn\|\|MnO_2_ | 102.3 (10 μA cm^−2^)  35.9 (100 μA cm^−2^) | 900 cycles, 62.8% (50 μA cm^−2^) | 141.9  135.4 | [22] |
| C\|\|PPYDBS | 10.6 (0.46 C)  – | – | 20.2  39.6 | [23] |
| NiSn\|\|LiMnO_2_ | 21.3 (2 C)  10.2 (20 C) | 200 cycles, 77% (20 C) | 44.9  6362.3 | [24] |
| Li_4_Ti_5_O_12_\|\|LiMn_2_O_4_ | 3.96 (5 C)  1.76 (300 C) | 100 cycles, 85% (5 C) | 9.45  2994.25 | [25] |

PPy: polypyrrole; MWCNTs: multiwalled carbon nanotubes; LDH: layered hydroxides; CC: carbon cloth; HCF-P: hexacyanoferrate-poly(3,4-ethylenedioxythiophene); PPYDBS: dodecylbenzenesulfonate-doped polypyrrole.

**Table S3.** The output high voltage comparison of our integrated MBs with the reported micro electrochemical energy storage devices

| **System** | **Size of a single device (mm^2^)** | **Integrated device number** | **Output voltage (V)** | **References** |
| --- | --- | --- | --- | --- |
| Photolithographic LVP\|\|LVP MBs | 2.2275 | 63 | 182.7 | This work |
| Screen-printed graphene-based MSCs | ~23.04 | 130 | 104 | [26] |
| Screen-printed MXene-based MSCs | ~7.95 | 100 | 60 | [27] |
| Inkjet-printed MXene/PH1000-based MSCs | 9.95 | 60 | 36 | [28] |
| Photolithographic MXene-based MSCs (3D-printed electrolyte) | 1.8 | 334 | 200 | [29] |
| 3D-printed electrochemically exfoliated graphene-based MSCs | 3.252 | 55 | 192.5 | [30] |
| Photolithographic MXene-based MSCs (surface adhesive-directed electrolyte assembly) | 0.3575 | 72 | 190 | [31] |
| Photolithographic CNT-based MSCs | ~0.63 | 100 | 100 | [32] |
| Laser-induced graphene-based MSCs | ~7.26 | 209 | 209 | [33] |
| Electrohydrodynamic jet-printed activated carbon-based MSCs | 1.27 | 36 | 43.2 | [34] |
| Direct-ink-written MoS_2_-based MSCs | 9 | 12 | 24 | [35] |
| Laser direct written graphite\|\|graphite MBs | ~18 | 36 | 180 | [36] |

**References**

1. Tong J, Su A, Ma T *et al.* Boosting low temperature performance of lithium ion batteries at −40°C using a binary surface coated Li_3_V_2_(PO_4_)_3_ cathode material. *Adv Funct Mater* 2024; **34**: 2310934.

2. Zheng S, Wu Z-S, Zhou F *et al.* All-solid-state planar integrated lithium ion micro-batteries with extraordinary flexibility and high-temperature performance. *Nano Energy* 2018; **51**: 613-20.

3. Zheng S, Huang H, Dong Y *et al.* Ionogel-based sodium ion micro-batteries with a 3D Na-ion diffusion mechanism enable ultrahigh rate capability. *Energy Environ Sci* 2020; **13**: 821-9.

4. Fu P, Zhao Y, Dong Y *et al.* Low temperature solid-state synthesis routine and mechanism for Li_3_V_2_(PO_4_)_3_ using LiF as lithium precursor. *Electrochim Acta* 2006; **52**: 1003-8.

5. Chen Z, Dai C, Wu G *et al.* High performance Li_3_V_2_(PO_4_)_3_/C composite cathode material for lithium ion batteries studied in pilot scale test. *Electrochim Acta* 2010; **55**: 8595-9.

6. Wang L, Jiang X, Li X *et al.* Rapid preparation and electrochemical behavior of carbon-coated Li_3_V_2_(PO_4_)_3_ from wet coordination. *Electrochim Acta* 2010; **55**: 5057-62.

7. Kotobuki M, Suzuki Y, Munakata H *et al.* Fabrication of three-dimensional battery using ceramic electrolyte with honeycomb structure by sol–gel process. *J Electrochem Soc* 2010; **157**: A493.

8. Pearse A, Schmitt T, Sahadeo E *et al.* Three-dimensional solid-state lithium-ion batteries fabricated by conformal vapor-phase chemistry. *ACS Nano* 2018; **12**: 4286-94.

9. Zhu M, Wang Z, Li H *et al.* Light-permeable, photoluminescent microbatteries embedded in the color filter of a screen. *Energy Environ Sci* 2018; **11**: 2414-22.

10. Shi J, Wang S, Chen X *et al.* An ultrahigh energy density quasi-solid-state zinc ion microbattery with excellent flexibility and thermostability. *Adv Energy Mater* 2019; **9**: 1901957.

11. Bi J, Zhang J, Giannakou P *et al.* A highly integrated flexible photo-rechargeable system based on stable ultrahigh-rate quasi-solid-state zinc-ion micro-batteries and perovskite solar cells. *Energy Storage Mater* 2022; **51**: 239-48.

12. Li X, Chen F, Zhao B *et al.* Ultrafast synthesis of metal-layered hydroxides in a dozen seconds for high-performance aqueous Zn (micro-) battery. *Nano-Micro Lett* 2023; **15**: 32.

13. Hao Z, Xu L, Liu Q *et al.* On-chip Ni–Zn microbattery based on hierarchical ordered porous Ni@Ni(OH)_2_ microelectrode with ultrafast ion and electron transport kinetics. *Adv Funct Mater* 2019; **29**: 1808470.

14. Sun P, Li X, Shao J *et al.* High-performance packaged 3D lithium-ion microbatteries fabricated using imprint lithography. *Adv Mater* 2021; **33**: 2006229.

15. Yang W, Xu L, Luo W *et al.* Rechargeable zinc-ammonium hybrid microbattery with ultrahigh energy and power density. *Matter* 2023; **6**: 3006-20.

16. Li Y, Zhu M, Bandari VK *et al.* On-chip batteries for dust-sized computers. *Adv Energy Mater* 2022; **12**: 2103641.

17. Li Y, Zhu M, Karnaushenko DD *et al.* Microbatteries with twin-swiss-rolls redefine performance limits in the sub-square millimeter range. *Nanoscale Horiz* 2023; **8**: 127-32.

18. Dokko K, Sugaya J-i, Nakano H *et al.* Sol–gel fabrication of lithium-ion microarray battery. *Electrochem Commun* 2007; **9**: 857-62.

19. Yoshima K, Munakata H, Kanamura K. Fabrication of micro lithium-ion battery with 3D anode and 3D cathode by using polymer wall. *J Power Sources* 2012; **208**: 404-8.

20. Kutbee AT, Bahabry RR, Alamoudi KO *et al.* Flexible and biocompatible high-performance solid-state micro-battery for implantable orthodontic system. *npj Flexible Electron* 2017; **1**: 7.

21. Jia B, Zhang C, Liu M *et al.* Integration of microbattery with thin-film electronics for constructing an integrated transparent microsystem based on InGaZnO. *Nat Commun* 2023; **14**: 5330.

22. Li P, Yang Z, Li C *et al.* Swimmable micro-battery for targeted power delivery. *Adv Funct Mater* 2024; **34**: 2312188.

23. Min H-S, Park BY, Taherabadi L *et al.* Fabrication and properties of a carbon/polypyrrole three-dimensional microbattery. *J Power Sources* 2008; **178**: 795-800.

24. Ning H, Pikul JH, Zhang R *et al.* Holographic patterning of high-performance on-chip 3D lithium-ion microbatteries. *Proc Natl Acad Sci* 2015; **112**: 6573-8.

25. Li W, Christiansen TL, Li C *et al.* High-power lithium-ion microbatteries from imprinted 3D electrodes of sub-10 nm LiMn_2_O_4_/Li_4_Ti_5_O_12_ nanocrystals and a copolymer gel electrolyte. *Nano Energy* 2018; **52**: 431-40.

26. Shi X, Pei S, Zhou F *et al.* Ultrahigh-voltage integrated micro-supercapacitors with designable shapes and superior flexibility. *Energy Environ Sci* 2019; **12**: 1534-41.

27. Zheng S, Wang H, Das P *et al.* Multitasking MXene inks enable high-performance printable microelectrochemical energy storage devices for all-flexible self-powered integrated systems. *Adv Mater* 2021; **33**: 2005449.

28. Ma J, Zheng S, Cao Y *et al.* Aqueous MXene/PH1000 hybrid inks for inkjet-printing micro-supercapacitors with unprecedented volumetric capacitance and modular self-powered microelectronics. *Adv Energy Mater* 2021; **11**: 2100746.

29. Wang S, Li L, Zheng S *et al.* Monolithic integrated micro-supercapacitors with ultra-high systemic volumetric performance and areal output voltage. *Natl Sci Rev* 2022; **10**: nwac271.

30. Zhang L, Qin J, Das P *et al.* Electrochemically exfoliated graphene additive-free inks for 3D printing customizable monolithic integrated micro-supercapacitors on a large scale. *Adv Mater* 2024; **36**: 2313930.

31. Wang S, Zheng S, Shi X *et al.* Monolithically integrated micro-supercapacitors with high areal number density produced by surface adhesive-directed electrolyte assembly. *Nat Commun* 2024; **15**: 2850.

32. Laszczyk KU, Kobashi K, Sakurai S *et al.* Lithographically integrated microsupercapacitors for compact, high performance, and designable energy circuits. *Adv Energy Mater* 2015; **5**: 1500741.

33. Li X, Cai W, Teh KS *et al.* High-voltage flexible microsupercapacitors based on laser-induced graphene. *ACS Appl Mater Interfaces* 2018; **10**: 26357-64.

34. Lee K-H, Lee S-S, Ahn DB *et al.* Ultrahigh areal number density solid-state on-chip microsupercapacitors via electrohydrodynamic jet printing. *Sci Adv* 2020; **6**: eaaz1692.

35. Lee K-H, Kim S-W, Kim M *et al.* Folding the energy storage: beyond the limit of areal energy density of micro-supercapacitors. *Adv Energy Mater* 2023; **13**: 2204327.

36. Liu Q, Zhang G, Chen N *et al.* The first flexible dual-ion microbattery demonstrates superior capacity and ultrahigh energy density: small and powerful. *Adv Funct Mater* 2020; **30**: 2002086.
